# Release of GTP Exchange Factor Mediated Down-Regulation of Abscisic Acid Signal Transduction through ABA-Induced Rapid Degradation of RopGEFs

**DOI:** 10.1371/journal.pbio.1002461

**Published:** 2016-05-18

**Authors:** Zixing Li, Rainer Waadt, Julian I. Schroeder

**Affiliations:** 1 Division of Biological Sciences, Cell and Developmental Biology Section, University of California San Diego, La Jolla, California, United States of America; 2 Institute of Plant Physiology and Ecology, Shanghai Institutes for Biological Sciences, Chinese Academy of Sciences, Shanghai, China; Duke University, UNITED STATES

## Abstract

The phytohormone abscisic acid (ABA) is critical to plant development and stress responses. Abiotic stress triggers an ABA signal transduction cascade, which is comprised of the core components PYL/RCAR ABA receptors, PP2C-type protein phosphatases, and protein kinases. Small GTPases of the ROP/RAC family act as negative regulators of ABA signal transduction. However, the mechanisms by which ABA controls the behavior of ROP/RACs have remained unclear. Here, we show that an *Arabidopsis* guanine nucleotide exchange factor protein RopGEF1 is rapidly sequestered to intracellular particles in response to ABA. GFP-RopGEF1 is sequestered via the endosome-prevacuolar compartment pathway and is degraded. RopGEF1 directly interacts with several clade A PP2C protein phosphatases, including ABI1. Interestingly, RopGEF1 undergoes constitutive degradation in *pp2c* quadruple *abi1/abi2/hab1/pp2ca* mutant plants, revealing that active PP2C protein phosphatases protect and stabilize RopGEF1 from ABA-mediated degradation. Interestingly, ABA-mediated degradation of RopGEF1 also plays an important role in ABA-mediated inhibition of lateral root growth. The presented findings point to a PP2C-RopGEF-ROP/RAC control loop model that is proposed to aid in shutting off ABA signal transduction, to counteract leaky ABA signal transduction caused by “monomeric” PYL/RCAR ABA receptors in the absence of stress, and facilitate signaling in response to ABA.

## Abstract 2

The hormone abscisic acid causes rapid re-localization and degradation of a guanine nucleotide exchange-factor, RopGEF1, suggesting a control-loop model that inhibits “leaky” signaling and facilitates the abscisic acid response.

## Author Summary

The plant hormone abscisic acid (ABA) is critical to plant development and stress responses. The ABA signaling cascade is comprised of ABA receptors, negatively regulating PP2C protein phosphatases and transducing protein kinases. Biochemical assays have indicated that certain ABA receptors constitutively bind to and inhibit PP2C protein phosphatases even without ABA. This finding has suggested that leaky receptor signal transduction in the absence of ABA could occur. Small GTPases named ROPs are negative regulators of ABA signal transduction and maintain PP2C protein phosphatase activity. However, whether and how the ABA signal can remove the inhibition of ABA signaling by ROPs remains elusive. The GTP exchange factor, RopGEF1, is an activator of ROP small GTPases. We show that the subcellular localization of RopGEF1 rapidly changes in response to ABA. RopGEF1 is sequestered via the endosome-prevacuolar compartment pathway and is degraded. Interestingly, we have found that ABI1, a PP2C protein phosphatase, directly interacts with RopGEF1. Moreover, PP2Cs, which are active in the absence of ABA, protect the protein stability of RopGEF1. These findings point to a RopGEF1-ROP-ABI1 control loop model that could protect against leaky receptor signaling, and which facilitates ABA signal transduction when ABA is produced in response to stress.

## Introduction

Abscisic acid (ABA) is a phytohormone that protects plants against abiotic stress and is involved in seedling development. In response to abiotic stress conditions, ABA concentrations rise in plant cells [1–3]. ABA can be perceived by a group of soluble “PYL/RCAR” ABA receptor proteins, which upon ABA binding lead to formation of ABA-PYL/RCAR-PP2C complexes that in turn inhibit PP2C protein phosphatase activity [4,5]. This releases PP2C-mediated inhibition of the downstream SnRK2 protein kinases [6,7]. Subsequently, SnRK2 protein kinases are activated and phosphorylate downstream transcription factors and ion channels to trigger ABA responses [1,3,8].

Recent structural and biochemical studies revealed that 14 *Arabidopsis* PYL/RCARs can be subdivided into two groups: those with a higher probability of forming PYL/RCAR dimers and those with a thermodynamically favored monomeric state. Monomeric PYL/RCARs bind to PP2Cs and may downregulate PP2C activity even in the absence of the ABA ligand [9,10]. Theoretically, this constitutive receptor activity would cause “leaky” ABA signal transduction [10]. Diverse receptor-mediated signal transduction mechanisms in eukaryotes include specific proteins that can shut off signaling in the absence of the stimulus, e.g., [11–13]. However, molecular mechanisms that protect against leaky ABA signal transduction remain unknown. Reconstitution studies of ABA signal transduction have demonstrated the fundamental roles of a set of core ABA signaling components PYL/RCAR-PP2C-SnRK/CDPKs [14–17]. Studies suggest that additional factors function in ABA signal transduction [18–22]. The linkage of some factors with core ABA signal transduction components needs to be further addressed.

RopGEFs are plant-specific “PRONE” (plant-specific *ROP* nucleotide exchanger)-domain-containing guanine nucleotide exchange factors [23,24]. The *Arabidopsis* genome encodes 14 RopGEFs with a high degree of sequence similarity, particularly for the residues that are involved in catalyzing GDP/GTP exchange [24]. Insights from the crystal structures of ROP-GDP-PRONE ternary intermediates and ROP-PRONE binary complexes revealed the molecular mechanism of activation of the small GTP-binding proteins in plants by RopGEFs through promoting exchange of GDP for GTP [25,26]. Besides activation of ROP/RACs, recent studies have suggested that RopGEFs also act as a bridge in the linkage of signal transduction from receptor-like kinases (RLKs) to ROP/RACs. The FERONIA RLK was shown to function as an upstream regulator of ROP/RAC signaling likely by interacting with RopGEFs to mediate auxin effects on root hair growth [27]. Another receptor-like kinase, AtPRK2, physically binds to and phosphorylates RopGEF1 in the C terminal region, which in turn promotes ROP1 activation during pollen tube growth [28].

In previous studies, it was demonstrated that ROP/RAC small GTPases act as negative regulators of ABA signal transduction [29,30], and ROP11 can directly bind to the type 2C protein phosphatase ABI1 and protect ABI1 protein phosphatase activity from inhibition by RCAR1/PYL9 [31]. Furthermore, ABA did not affect the mRNA or protein levels of ROP11 in plants [32]. However, the mechanisms by which ABA regulates activity of the ROP11 GTPase remain unknown. We investigated whether ABA may control the behavior of ROPs through affecting the ROP activators RopGEFs. Here, we show that ABA signal transduction directly mediates the removal of RopGEF1 via relocation to and degradation in vacuoles, thus enabling robust ABA signal transduction. Moreover, PP2C phosphatases directly interact with RopGEF1 and inhibit the degradation of RopGEF1 in the absence of ABA, thus providing a mechanism to ensure shutting off of ABA signal transduction. Based on our results, a GEF-ROP-PP2C control loop model is suggested that prohibits leaky ABA signal transduction and that functions in modulation of ABA signaling strength.

## Results

### RopGEF1 Relocates to Intracellular Particles in Response to ABA

To study the effects of ABA signal transduction on RopGEF1 protein (from here on “GEF1”), we first investigated the subcellular localization of GEF1 in the absence and presence of exogenous ABA. We expressed GFP-GEF1, GEF1-GFP, and GEF1-mCherry fusion proteins in *Nicotiana benthamiana* epidermal cells. All three constructs showed similar fluorescence signal patterns (S1A–S1C Fig). We then generated GFP-GEF1 overexpression lines in *Arabidopsis* wild-type plants. These transgenic lines exhibited swollen root hairs similar to those found upon overexpression of GEF1 only or overexpression of a constitutively active ROP11 (S1F Fig) [33], a downstream substrate of GEF1 [34]. These observations indicated that the GFP-GEF1 construct is functional in *Arabidopsis* plants.

**Fig 1:**
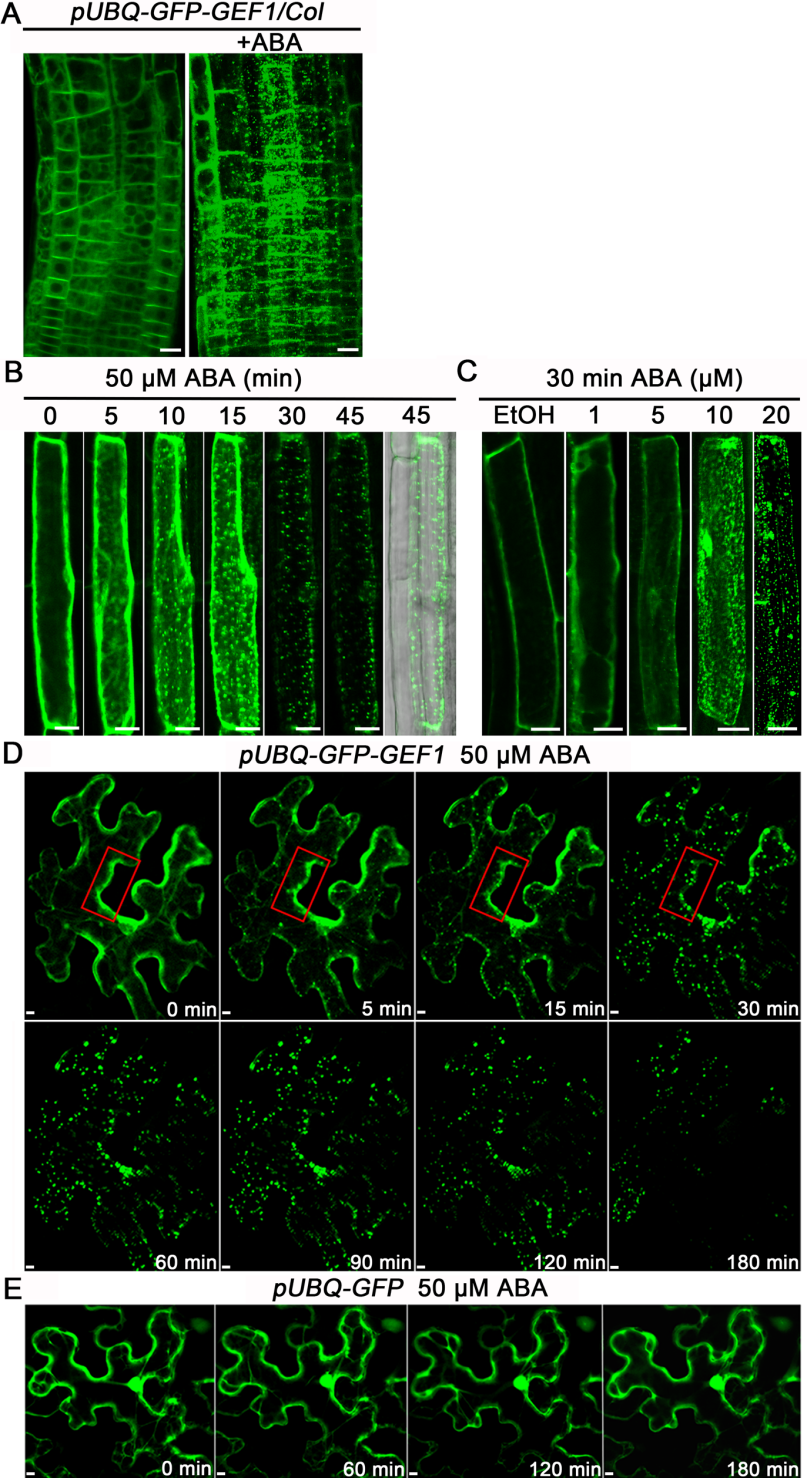
ABA treatment changes the subcellular localization of GFP-GEF1. (A) Subcellular localization of GFP-GEF1 in epidermal cells of the root elongation zone of *Arabidopsis* without ABA treatment (left) or treated with 50 μM ABA for 30 min (right). (B) Time-lapse confocal images of a representative *Arabidopsis* root epidermal cell expressing GFP-GEF1 in 4-day old seedlings exposed to 50 μM ABA. Right: GFP and bright field merged image indicates that 45 min of ABA treatment does not disrupt cell integrity. (C) Dose-dependent effect of ABA-mediated relocation of GFP-GEF1 in *Arabidopsis* root epidermal cells in 4-day old seedlings 30 min after the indicated treatments. 0.1% (v/v) ethanol (EtOH) treatment as a control. (D) Time-lapse confocal images of a representative *Nicotiana benthamiana* leaf epidermal cell expressing GFP-GEF1 exposed to 50 μM ABA. The red boxes provide an example illustrating the observed changes in fluorescence. (E) Control time-lapse confocal images of a representative *N*. *benthamiana* leaf epidermal cell expressing GFP only exposed to 50 μM ABA. Confocal images were captured and are shown with the same parameters for each panel within B, D, and E. Results are representative confocal images from *n* ≥ 3 separate experiments, and >5 independent cell images were captured per experiment. Scale bars 10 μm.

Confocal microscopy revealed that in the absence of exogenous ABA, the GFP-GEF1 signal was largely located in the cytosol and cell periphery (Fig 1A left and S1A–S1C Fig), where GEF1-mCherry fluorescence signal was visible following NaCl-induced plasmolysis (S1C Fig) and GFP-GEF1 fluorescence partially overlapped with the lipophilic dye FM4-64 signal, which stains the plasma membrane (S1E Fig). Surprisingly, after application of ABA, numerous intracellular fluorescent particles appeared in *Arabidopsis* root epidermal cells (Fig 1A right). Further analyses showed that formation of these particles was time- and ABA-dose-dependent (Fig 1B and 1C). Experiments investigating different hormone stimuli including auxin, GA, ethylene, JA, and brassinosteroid demonstrated that this effect was specific to ABA relative to these hormones (S2A Fig).

To better visualize this ABA-mediated formation of intracellular particles, we examined the subcellular localization of GFP-GEF1 in *N*. *benthamiana* leaves treated with ABA. Time-course confocal microscopy revealed that within 5 min of ABA treatment, GFP-GEF1 moved from the cell periphery and gradually accumulated in numerous particles (Fig 1D). Subsequently, the fluorescence intensity was substantially reduced over a time period of 30–180 min (Fig 1B and 1D). In comparison, the subcellular localization and fluorescence signal intensity of GFP alone was not altered under the same ABA treatment (Fig 1E). Furthermore, GFP-GEF1 driven by the *GEF1* promoter also formed particles in response to ABA (S2D Fig). In controls without ABA addition, 3 h of microscopy did not cause GFP-GEF1 to form particles (S2E Fig).

### ABA Treatment Causes RopGEF1 Relocation to the Prevacuolar Compartment

**Fig 2:**
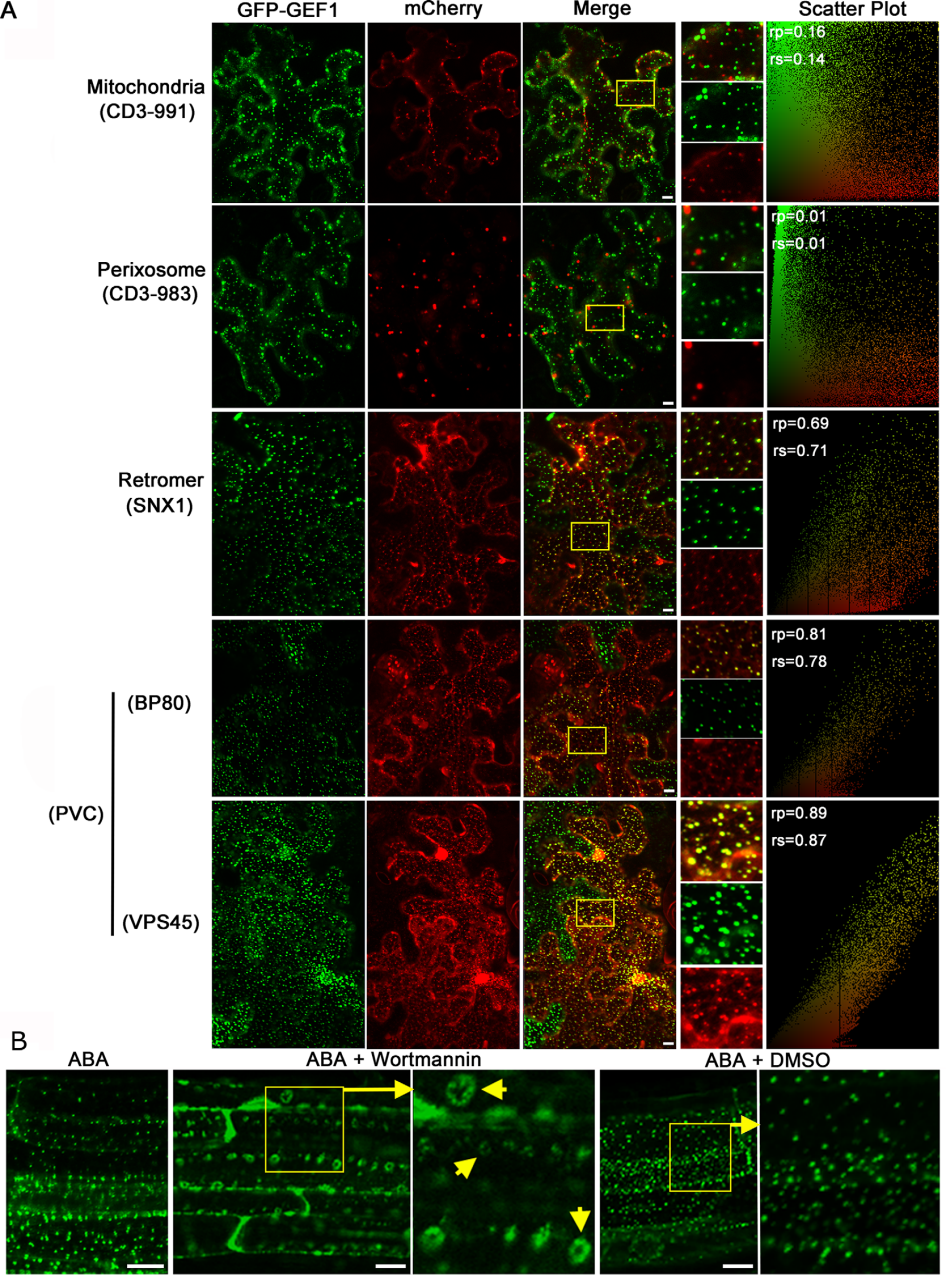
GFP-GEF1 is relocated to the prevacuolar compartment (PVC) in response to ABA treatment. (A) Co-localization assays of GFP-GEF1 with the indicated organelle markers in *N*. *benthamiana* leaf epidermal cells in response to ABA. GFP-GEF1 was co-expressed with mCherry-labeled organelle markers in *N*. *benthamiana* leaf epidermal cells. *N*. *benthamiana* leaves were treated with 50 μM ABA for 1 h before confocal imaging. Co-localization is visible as yellow dots in the merged images (Merge). Organelle marker names are listed in parentheses on the left. PVC, pre-vacuolar compartment. Representative images are shown of three independent co-localization experiments. (see Methods). Yellow boxes indicate approximate regions amplified to the right of merged images. Levels of co-localization for yellow boxed regions are depicted in relative intensity (*x-*, *y*-axes) scatter plots. Values of the linear Pearson correlation coefficient (rp) and the non-linear Spearman’s rank (rs) correlation coefficient were calculated and are given in the upper left corner of scatter plots. (B) Confocal images of the subcellular localization of GFP-GEF1 in response to the indicated treatments. 4-day-old *Arabidopsis* seedlings overexpressing GFP-GEF1 incubated with 50 μM ABA only (left), ABA plus 20 μM Wortmannin (PI3K inhibitor, disrupts protein trafficking into PVC) (middle), or ABA plus 0.1% (v/v) DMSO (Wortmannin solvent) (right) for 1 h before confocal imaging. Yellow boxes indicate regions of magnified images to the right of boxed images. Short yellow arrows point to ring-like structures typically induced by Wortmannin treatment. Scale bars 10 μm.

Experiments were carried out to determine the identity of the ABA-induced GFP-GEF1 particles. We investigated the co-localization of GFP-GEF1 in *N*. *benthamiana* leaves with mCherry labeled organelle markers [35]. No co-localization or sparse overlap was observed with mitochondrial, peroxisomal, ER, and *cis*-Golgi markers (Figs 2A and S3A), indicating that GFP-GEF1 proteins were not sequestered into these endomembrane systems in response to ABA. In comparison, overlap of the GFP and mCherry signals was observed in co-localization analysis with the retromer marker SNX1 [36] and the prevacuolar compartment (PVC) markers BP-80 [37] and VPS45 (Fig 2A) [38].

Reminiscent of recent studies showing that the plasma membrane proteins, FLAGELLIN SENSITIVE2 (FLS2), the brassinosteroid receptor BRI1, and the auxin efflux carrier PIN2 are internalized rapidly from the plasma membrane and sequentially delivered into the vacuole via the late endosome [39–41], we speculated that GFP-GEF1 proteins were also subjected to this process in response to ABA. In line with this hypothesis, ABA-induced GFP-GEF1 particle formation was sensitive to Wortmannin (Fig 2B), a phosphatidylphosphate-3-kinase (PI3K) inhibitor that interferes with vesicle trafficking from the plasma membrane to the prevacuolar compartment [42,43]. GFP-GEF1 particles accumulated into typical Wortmannin-induced intracellular ring-like structures (Fig 2B) [44,45]. In addition, we observed that ABA-induced GFP-GEF1 particles partially co-localized with an endosomal (LE) marker ARA7 (S3A Fig) [46]. In response to stimulation by a combination of ABA and Brefeldin A (BFA), an inhibitor of endosomal transport [47], GFP-GEF1 particles aggregated into large blocks (S3C Fig), known as BFA bodies [48]. Furthermore, subcellular localization of GFP-GEF1 did not showed significant change in response to Wortmannin or BFA treatment, respectively (S3C Fig). The above analysis suggested that GFP-GEF1 proteins are relocated to the prevacuolar compartment via an endosome–prevacuolar compartment pathway in response to ABA.

### RopGEF1 Is Degraded in Response to ABA

**Fig 3:**
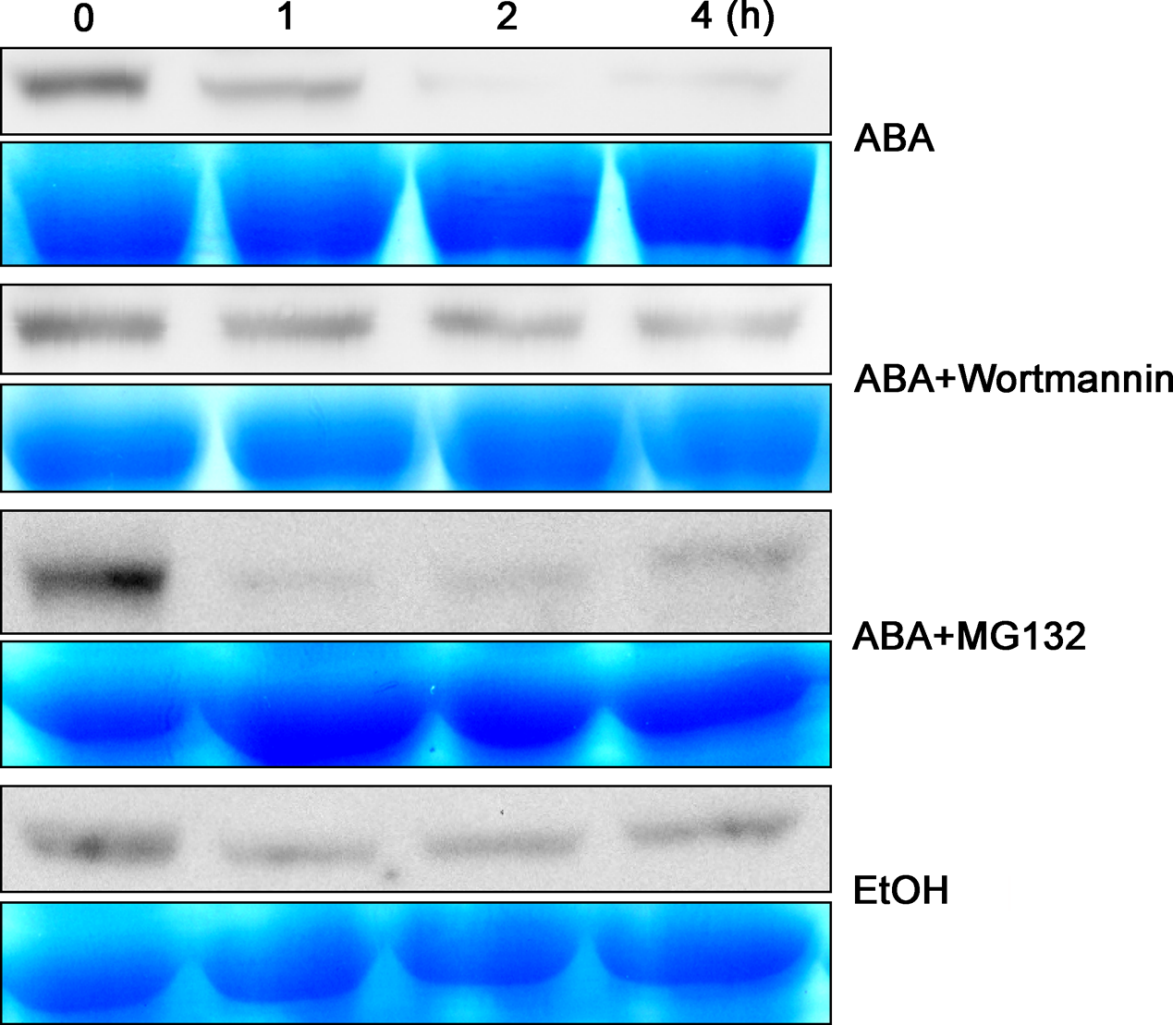
GEF1 protein is degraded in response to ABA treatment. Western blot analyses of GFP-GEF1 protein levels in response to the indicated treatments. Ten-day-old *pUBQ-GFP-GEF1 Arabidopsis* seedlings were immersed in 1/2 MS liquid medium for 1 h, then transferred into 1/2 MS medium supplemented with 50 μM ABA or 50 μM ABA plus 20 μM Wortmannin, and 0.1% (v/v) EtOH for the indicated durations. For MG132 treatment, seedlings were immersed in 1/2 MS medium with 50 μM MG132 for 2 h, then transferred in 1/2 MS medium with 50 μM ABA plus 50 μM MG132 for the indicated durations. Total protein extracts (20 μg) were subjected to immunoblot analysis with GFP antibody. Coomassie blue staining of SDS-polyacrylamide gel (SDS-PAGE) was used as a loading control.

Proteins in the prevacuolar compartment are further transported into vacuoles through membrane fusion and are subsequently subjected to either storage or degradation [49]. To trace the fate of GFP-GEF1, we carried out western blot analyses to detect GFP-GEF1 fusion protein abundance in response to ABA in transgenic *Arabidopsis* plants. Immunoblot analyses revealed that GFP-GEF1 protein levels were rapidly and substantially reduced in response to ABA treatment (Fig 3), but only slightly altered in response to the control ethanol (EtOH) stimulus (solvent used for ABA) and combined ABA and Wortmannin (Fig 3). Moreover, ABA-mediated degradation of GFP-GEF1 was still evident in the presence of the proteasome inhibitor MG132 (Fig 3), suggesting no major role of the proteasome in this degradation response. Furthermore, confocal microscopic observations indicated that ABA-mediated GFP-GEF1 particles showed co-localization with the vacuolar marker γ-TIP (S3B Fig) [50,51]. Taken together, we concluded that GFP-GEF1 is sequestered to vacuoles and is degraded in response to ABA.

### RopGEF1 Directly Interacts with Several PP2C Phosphatases

**Fig 4:**
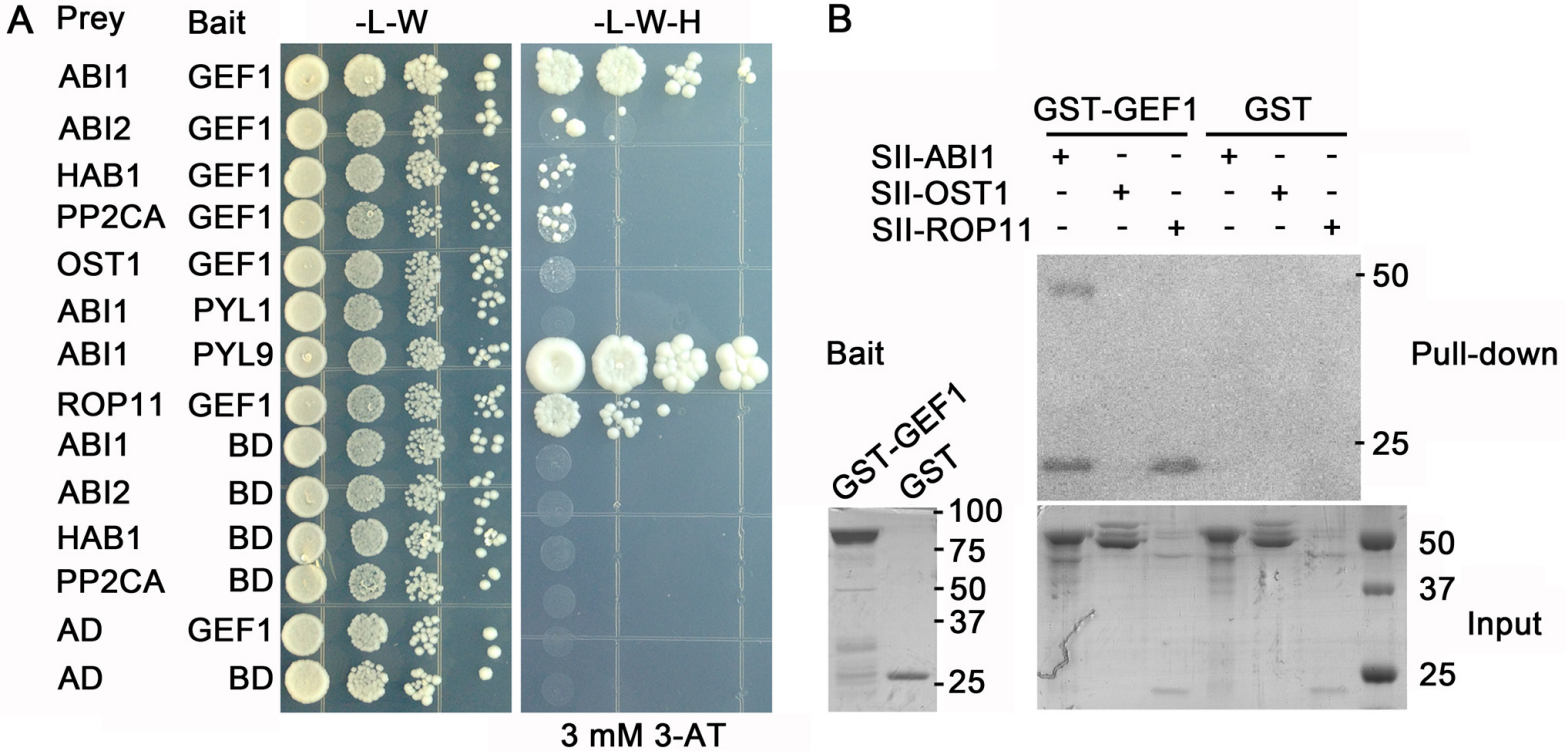
GEF1 directly interacts with *Arabidopsis* type 2C protein phosphatase ABI1. (A) Yeast-two-hybrid (Y2H) assay of interactions of GEF1 with the indicated PP2Cs. The indicated construct combinations were co-transformed into the yeast strain pJ69-4A. Transformants were grown on-L-W control plates (left) for 3 d and -L-W-H (lacking Leucine, Tryptophan, and Histidine) selective plates with 3 mM 3-amino-1,2,4-triazole (3-AT) (right) for 6 d. The interactions of ABI1/PYL9 and ROP11/GEF1 were used as positive controls, and ABI1/PYL1 without ABA as negative control. (B) In vitro binding assay of GEF1 and ABI1. Each input protein was incubated with Glutathione Sepharose beads containing GST-GEF1 or GST control protein, followed by GST-GEF1 and GST affinity purification and subsequent immunoblotting with anti-Strep-II antibody. Bait (left): Coomassie brilliant blue staining of purified bait protein GST-GEF1 and GST; Pull-down: western blot of pull-down with Gluothathione Sepharose and probed with anti-Strep-II antibody; Input: Coomassie brilliant blue staining of recombinant input proteins StrepII-ABI1, StrepII-OST1, and StrepII-ROP11. ROP11/GEF1 and OST1/GEF1 binding were used as positive and negative controls, respectively.

We pursued yeast-two-hybrid (Y2H) experiments with GEF1 as bait in search of key ABA signal transduction factors that might participate in ABA-mediated degradation of GEF1. The type 2C protein phosphatase (PP2C) ABI1 was identified as a candidate GEF1 interactor. Interaction between ABI1 and GEF1 was observed in Y2H assays (Fig 4A). Known interactions between ABI1 and RCAR1/PYL9 [4] or GEF1 and ROP11 [34] served as positive controls (Fig 4A). In comparison with ABI1, other PP2Cs including ABI2, HAB1, and PP2CA exhibited weak interactions with GEF1 (Fig 4A). However, no reproducible interactions were observed for GEF1 and OST1, which is a direct substrate of ABI1 [6,52,53]. Lack of ABI1 and RCAR12/PYL1 interactions in the absence of ABA was used as a negative control (Fig 4A).

The putative interaction of ABI1 and GEF1 was further investigated in *in vitro* pull-down assays. Using GST-tagged GEF1 as bait, Strep-II tagged ABI1 and also ROP11 but not OST1 co-immunoprecipitated with GST-GEF1 (Fig 4B). Next, we pursued bimolecular fluorescence complementation (BiFC) assays to investigate putative interactions between GEF1 and PP2Cs in plant cells. Surprisingly, numerous fluorescent particles were detected in the cytosol (S4A Fig). Quantitative analyses of BiFC signals showed strong ABI1-GEF1 interactions and weak signals for GEF1 interaction with other clade A PP2Cs (S4A and S4B Fig), which correlated with observations from Y2H experiments (Fig 4A). In positive controls, strong BiFC signals for ROP11 and GEF1 interaction were observed, which appeared at the cell periphery (S4 Fig). Co-localization analyses with GFP-GEF1 and mCherry-ABI1 indicated that GEF1 and ABI1 localizations overlapped at the cell periphery without ABA treatment (S5A Fig). Furthermore, Y2H and Co-IP experiments showed that GEF1 also interacted with additional PP2Cs in the clade A PP2C phosphatase family, including HAI1 and AHG1 (S5B and S5C Fig).

### PP2C Phosphatases Protect RopGEF1 from ABA-Induced Degradation

**Fig 5:**
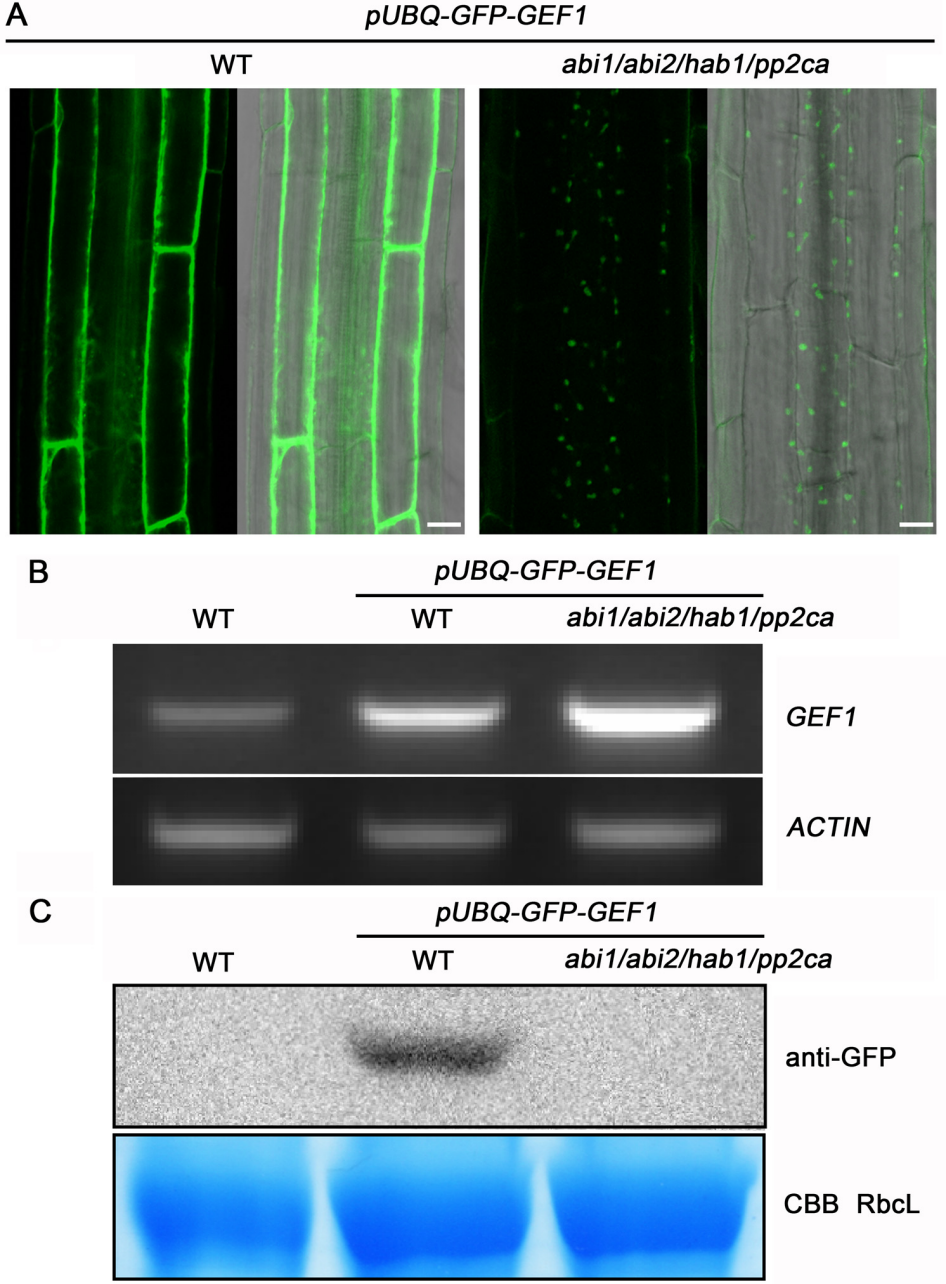
GFP-GEF1 is constitutively degraded in *abi1/abi2/hab1/pp2ca* quadruple mutant background. (A) Subcellular localization of GFP-GEF1 in root epidermal cells of wild type (left) and *abi1/abi2/hab1/pp2ca* background (right) in the absence of exogenous ABA. Confocal images were acquired and are shown with the same parameters except master gain: 700 (WT), 980 (*abi1abi2hab1pp2ca*). Scale bars: 10 μm. The depicted imaging experiments were repeated at least three times (see Methods). (B) RT-PCR analyses of *GEF1* transcripts in wild-type plants and *GFP-GEF1* overexpression lines in wild-type and *abi1/abi2/hab1/pp2ca* mutant background. RT-PCR for *ACTIN2* transcripts was used as loading control. (C) Western blot analyses of GFP-GEF1 protein levels with anti-GFP antibody in wild-type plants and *GFP-GEF1* overexpression lines in wild-type and *abi1/abi2/hab1/pp2ca* mutant background. Coomassie blue (CBB) stain of SDS-PAGE gel was used as a loading control. RbcL, RubisCO large subunit. Roots of 10-day-old seedlings grown on half MS medium were collected to extract total RNA (B) and protein (C).

We performed experiments to determine whether PP2Cs are involved in regulating the formation of intracellular GFP-GEF1 particles. We expressed the same *pUBQ-GFP-GEF1* construct as used in wild-type *Arabidopsis* plants for subcellular localization analyses (Fig 1A and 1B) in the *pp2c* quadruple mutant background *abi1/abi2/hab1/pp2ca* and investigated the subcellular localization of GFP-GEF1. Over 100 transgenic lines were checked, and all transgenic lines showed extremely weak GFP signals compared with those in the wild-type background (Fig 5A). RT-PCR and quantitative RT-PCR analyses showed that this is not because of a reduced transcript level of the GEF1 mRNA (Figs 5B and S8C). Interestingly, subcellular localization analyses of GFP-GEF1 showed fluorescence in intracellular particles of *abi1/abi2/hab1/pp2ca* root cells even without ABA treatment (Fig 5A).

To investigate the identity of these particles, transgenic GFP-GEF1/*abi1abi2hab1pp2ca* seedlings were subjected to Wortmannin treatment. Confocal microscopic observations indicated that these particles partially aggregated into ring-like structures 1 h after Wortmannin treatment (S6A Fig). After prolonged Wortmannin exposure of GFP-GEF1/*abi1abi2hab1pp2ca* plants, GFP-GEF1 fluorescence signals appeared in the cell periphery and perinuclear region (S6B Fig). This cellular localization pattern resembled that of subcellular localization GFP-GEF1 in the wild-type background in the absence of ABA (S1A–S1C Fig). These confocal microscopic analyses strongly suggested that GFP-GEF1 protein in *abi1/abi2/hab1/pp2ca* plants underwent constitutive degradation in the absence of added ABA. These findings suggested that the wild-type PP2Cs prohibit spontaneous degradation of GFP-GEF1. Furthermore, in newly emerging lateral roots, the intracellular particles were not degraded and instead aggregated into large intracellular fluorescent bodies (S6C Fig). These observations are consistent with the lack of the expression of the vacuolar TIP markers in these developing new *Arabidopsis* root tip cells that have been proposed to lack lytic vacuoles at this developmental stage [54,55].

We next investigated GFP-GEF1 protein levels in GFP-GEF1/*abi1abi2hab1pp2ca* plants by western blotting. Although GEF1 mRNA levels were abundant in GFP-GEF1/*abi1abi2hab1pp2ca* plants (Figs 5B and S8C), we could not detect a clear western blot signal, indicating the GFP-GEF1 protein levels are extremely low in the *abi1/abi2/hab1/pp2ca* quadruple mutant in contrast to wild-type plants (Fig 5C). Additional western blot experiments showed that GFP-GEF1 protein levels were measurably enhanced after 3 h of Wortmannin treatment (S6D and S6E Fig). Taken together, these data show that PP2C protein phosphatases interact with GEF1 (Figs 4, S4 and S5), and in wild-type plants prevent GFP-GEF1 degradation in the absence of ABA (Fig 5).

### RopGEF1 Is Epistatic to PP2C Phosphatases in ABA Signal Transduction Pathway

Previously, the small GTPase ROP11 in its active form was shown to directly bind to ABI1 and protect ABI1 protein phosphatase activity from inhibition by the ABA receptor RCAR1/PYL9 [31]. Given that GEFs activate ROPs through facilitating GDP/GTP exchange [25,26], we speculated that GEF1 plays a positive role in PP2C function. To dissect the genetic relevance between GEF1 and the PP2C protein phosphatases, we analyzed ABA-mediated phenotypes in GFP-GEF1 /*abi1abi2hab1pp2ca* overexpression plants.

**Fig 6:**
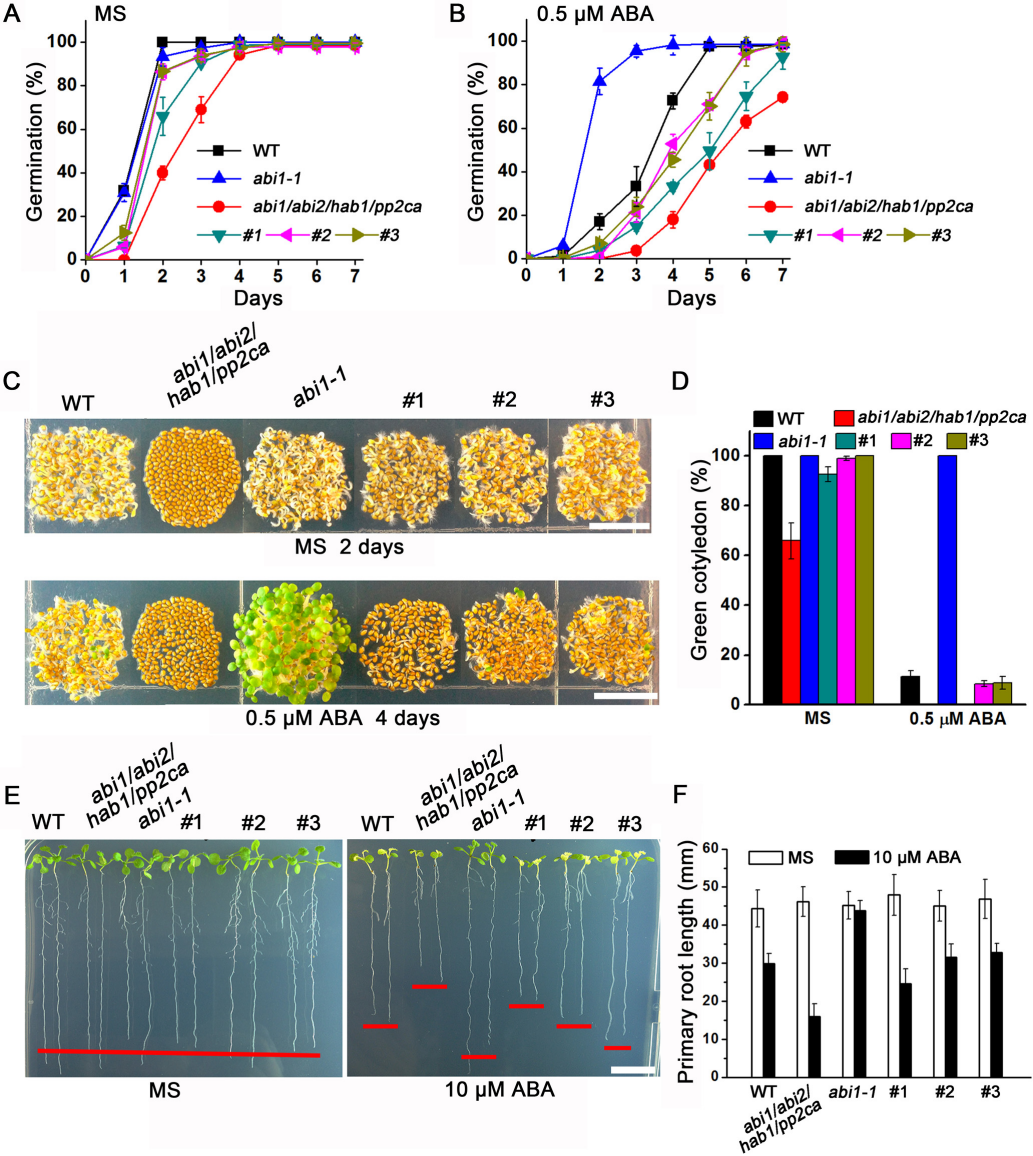
Overexpression of GFP-GEF1 in *abi1/abi2/hab1/pp2ca* background partially suppresses the ABA hypersensitive phenotype of *pp2c* quadruple mutant. (A,B) The percentage of seeds that germinated (radicle emergence) on 1/2 MS media (A) or supplemented with 0.5 μM ABA (B). Approximately 70 seeds of each genotype were sown and scored for radical emergence after 3 d of stratification. #1, #2, #3 represents three independent transgenic *GFP-GEF1/abi1abi2hab1pp2ca* overexpression lines. Error bars represent standard deviation. (C) Images of germinating seeds grown on 1/2 MS medium for 2 d (top row) or on 1/2 MS media supplemented with 0.5 μM ABA for 4 d (bottom row). Scale bars: 1 cm. (D) Percentage of seeds that developed green expanded cotyledons at day 6 after stratification. (E) Images of representative seedlings 5 d after the transfer of 4-day-old seedlings from 12/ MS media to plates lacking (left) or supplemented with 10 μM ABA (right). Seeds of each genotype were stratified for 10 d for synchronous germination. Red bars indicate approximate root lengths. Scale bars: 1 cm. (F) Quantification of ABA-mediated root growth inhibition. Twenty 4-day-old seedlings grown on 1/2 MS medium with similar primary root length were transferred onto 1/2 MS medium lacking or supplemented with 10 μM ABA. After 5 d of growth, images were taken and primary root length was measured with Image J software. Error bars represent SD.

We first examined ABA-mediated inhibition of seed germination and seedling establishment. After 3 d of stratification, *abi1/abi2/hab1/pp2ca* mutant seeds showed a late germination phenotype on 1/2 MS medium regardless of the presence of ABA (Fig 6A and 6B). In comparison, three independent *GFP-GEF1/abi1aib2hab1pp2ca* transgenic lines showed partial but not full restoration of seed germination and seedling establishment in the *pp2c* quadruple mutant background (Fig 6A–6F), which may be linked to the interaction of GEF1 with additional clade A PP2Cs (S5B and S5C Fig).

We then analyzed the responses of these genotypes in ABA-mediated inhibition of primary root elongation. To ensure simultaneous seed germination, seeds of all genotypes were stratified for 10 d. Seedlings with similar root lengths were transferred onto 1/2 MS plates supplemented with or without ABA. The results indicated that *GFP-GEF1/abi1abi2hab1pp2ca* seedlings were less sensitive to ABA-mediated inhibition of primary root growth than the *pp2c* quadruple mutant (Fig 6E and 6F). These phenotypic assays provide initial evidence for a genetic interaction between GEF1 and PP2Cs in ABA responses.

### RopGEFs Are Negative Regulators of the ABA Signal Transduction Pathway

**Fig 7:**
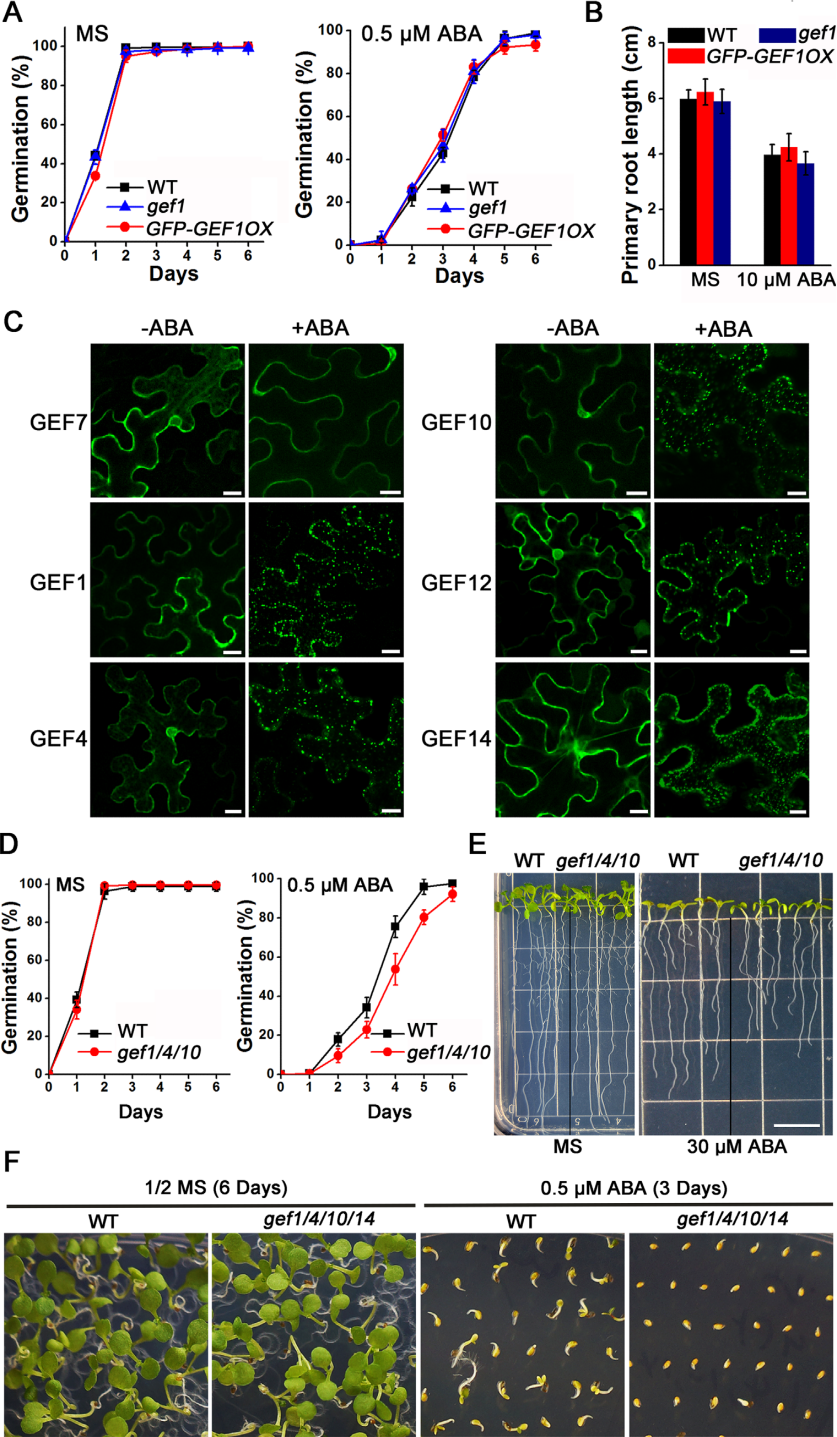
*gef1/4/10* triple mutant plants are hypersensitive to ABA treatment. (A) The percentage of seeds that germinated (radicle emergence) on 1/2 MS medium lacking (left) or supplemented with 0.5 μM ABA (right). Approximately 100 seeds of wild-type, *gef1* T-DNA insertion single mutant and *GFP-GEF1* overexpression transgenic lines were sown and scored for radical emergence for a time period of 6 d. (B) Quantification of ABA-mediated inhibition of primary root growth in wild type, *gef1* T-DNA insertion single mutant and *GFP-GEF1* overexpression transgenic lines. Thirty-two 4-day-old seedlings grown on 1/2 MS medium with similar primary root lengths were transferred onto 1/2 MS medium lacking or supplemented with 10 μM ABA. After 7 d of growth, images were taken and primary root length was measured with Image-J software. (C) Subcellular localization of the indicated RopGEFs in *N*. *benthamiana* leaves with or without ABA treatment. *N*. *benthamiana* leaves were treated with 50 μM ABA for 1 h before confocal imaging. Amplification factors for each panel were not identical and were set to visualize GFP fluorescence. Scale bars: 10 μm. (D) The germination percentage of wild-type and *gef1/4/10* triple mutant seeds on 1/2 MS medium lacking (left) or supplemented with 0.5 μM ABA (right). (E) Photograph of seedlings grown on 1/2 MS media supplemented with or without 30 μM ABA taken 6 d after transferring seedlings. Scale bar: 1 cm. (F) *gef1/4/10/14* quadruple mutant seeds showed enhanced sensitivity to ABA-mediated inhibition of seedling establishment. Images were taken 6 d (left) and 3 d (plus ABA, right) after stratification. Error bars represent standard deviation. A plate image of the same plate is shown in S11 Fig and includes *gef1/4/10* and *gef1/4/14* triple mutant seeds.

To pursue a more direct genetic investigation of GEF1 functions in ABA signaling, we examined ABA-related phenotypes in *GEF1* single knockout mutant and *GEF1* overexpression lines. However, we did not observe any differential responses to ABA- mediated inhibition of seed germination and primary root growth in these two genotypes compared with wild-type (Fig 7A and 7B). We speculated that this result was attributable to possible overlapping gene functions, considering the high sequence homology among members of the large GEF family. To circumvent possible redundancy, we pursued a generation of higher-order *gef* mutants. Considering the rapid GEF1 degradation in response to ABA, we assumed that other GEFs with similar functions as GEF1 in ABA signal transduction should be removed in the same manner in response to ABA. Based on this hypothesis, we examined the subcellular localization of all 14 members of the GEF family in the absence and presence of ABA. The results showed that like GEF1, GEF4, 10, 12, and 14 formed particles in response to ABA (Fig 7C). Other GEFs did not show ABA-induced intracellular particle formation (e.g., GEF7; Fig 7C). By analyzing ABA-mediated phenotypes in higher-order *gef* mutants, we found that a triple mutant *gef1/4/10* showed a slightly enhanced sensitivity to ABA-mediated inhibition of primary root elongation and seed germination compared to wild-type (Fig 7D and 7E), consistent with a previous report [56]. This slight hypersensitivity to ABA-mediated inhibition of seed germination was further enhanced in *gef1/4/10/14* quadruple mutant seed (Figs 7F and S11). The above analyses provided evidence to support that GEFs act as negative regulators of ABA responses.

### RopGEF1 Plays an Important Role in ABA-Mediated Inhibition of Lateral Root Growth

**Fig 8:**
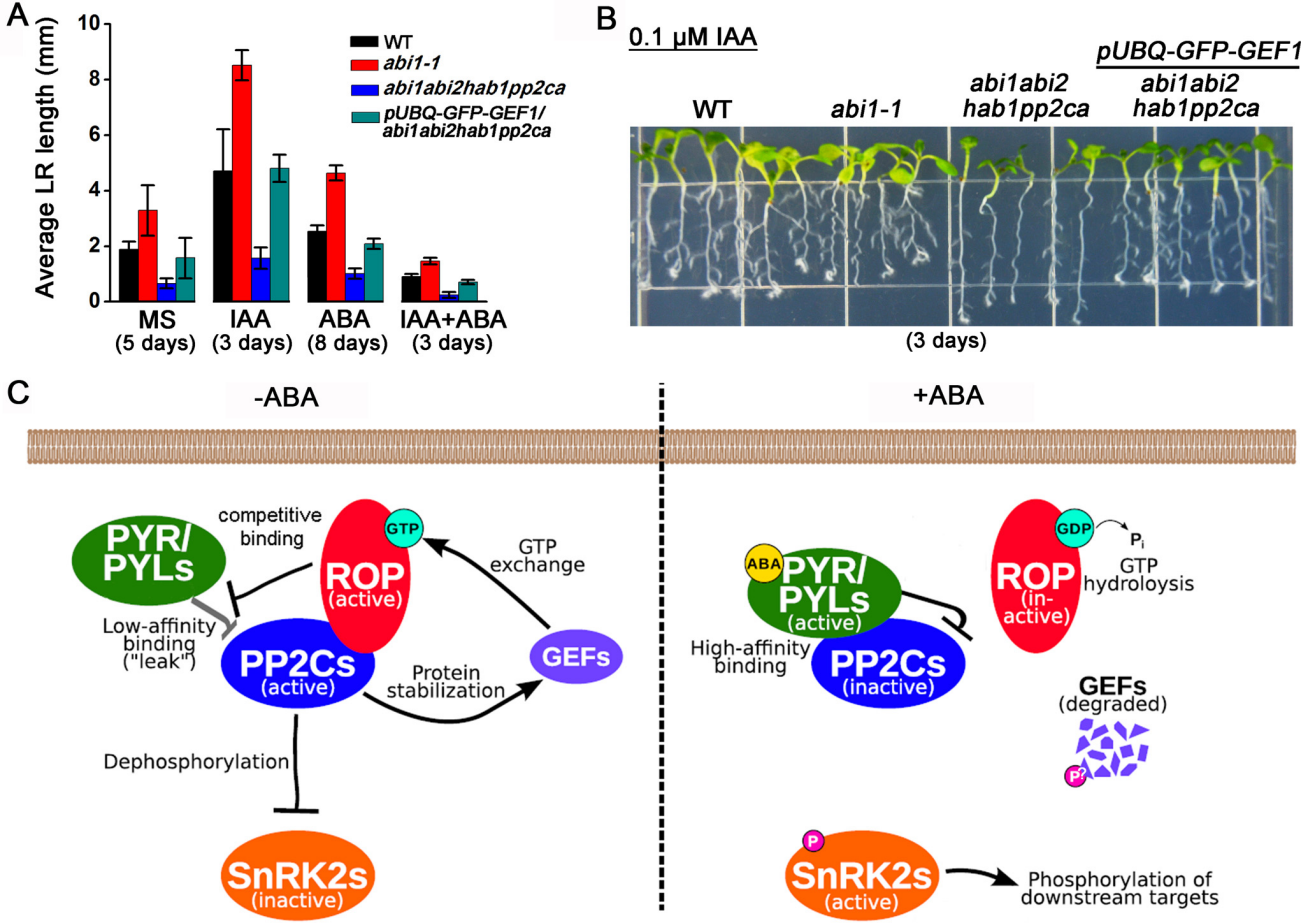
Overexpression of *GFP-GEF1* in *abi1/abi2/hab1/pp2ca* plants partly rescues lateral root growth deficiency of *pp2c* quadruple mutant. (A) Quantification of average lateral root (LR) length in wild-type, *abi1-1*, *abi1/abi2/hab1/pp2ca*, and *pUBQ-GFP-GEF1*/*abi1abi2hab1pp2ca* overexpression plants. Twenty 4-day-old seedlings grown on 1/2 MS medium with similar primary root lengths were transferred onto 1/2 MS medium or 1/2 MS medium supplemented with 0.1 μM IAA, 10 μM ABA, or 0.1 μM IAA plus 10 μM ABA (IAA was used here to accelerate lateral root growth). After the indicated times of growth on hormone-containing or control media, lateral roots were scanned and measured. Note that the bar graphs are from different time points to accurately resolve lateral root lengths for each condition (See Methods). Error bars represent standard deviation (*n* = 3). (B) Representative image showing lateral root growth of different seedling genotypes grown on 1/2 MS medium supplemented with 0.1 μM IAA. Image was taken 3 d after transferring 4-day-old seedlings to IAA-containing media. An image for similar experiments to those shown in (B) can be found in S7G Fig with 10 uM ABA added. (C) Working model for PP2C-RopGEF-ROP control loop in regulating ABA signal transduction. Arrows indicate activation, and bars indicate repression. Details are described in the text.

During exploration of the biological significance of ABA-mediated degradation of the GEF1 protein, we noticed that *abi1/abi2/hab1/pp2ca* mutant plants showed a strong reduction in lateral root growth on 1/2 MS medium (Figs 8A and S7E). The lateral root growth deficiency was also observed on MS medium supplemented with ABA, IAA, or both (Figs 8A, 8B, S7F and S7G) (IAA was used here to accelerate lateral root growth). Interestingly, overexpression of GFP-GEF1 partially rescued lateral root length and visible lateral root number defects of *abi1/abi2/hab1/pp2ca* mutant plants (Fig 8A and 8B, S7A–S7G Fig).

Given the reduction of lateral root length (Fig 8A and 8B) together with the low GEF1 protein abundance in *abi1/abi2/hab1/pp2ca* mutant plants (Fig 5A and 5C), we hypothesized that GEF1 together with other GEFs might have the ability to promote lateral root growth. To examine this hypothesis, we studied lateral root growth in wild-type, *pyr1/pyl1/2/4* ABA receptor quadruple mutant and *GFP-GEF1/*WT and *GFP-GEF1/pyr1pyl124* overexpression lines. We found that both *GFP-GEF1/*WT and *GFP-GEF1/pyr1pyl124* transgenic lines showed longer average lateral root lengths compared with those in the control wild type (*p* = 0.002, two-sample *t* test, Origin) and *pyr1/pyl1/2/4* mutant lines *(p =* 0.02, two-sample *t* test, Origin), respectively (S8A Fig). This result supported that GEF1 has an ability to promote lateral root growth. Interestingly, in the presence of ABA in wild type, the longer lateral root phenotype in *GFP-GEF1/*WT plants was repressed (S8B Fig, *p* = 0.26, two-sample *t* test, Origin). In comparison, lateral root length in *GFP-GEF1/pyr1pyl124* was longer than those in *pyr1/pyl1/2/4* mutant plants in the presence of ABA (S8B Fig, *p* = 0.03, two-sample *t* test, Origin). These data are consistent with a model in which ABA contributes to regulation of RopGEF1-mediated lateral root growth. Furthermore, we also examined lateral root growth in several higher-order *gef* mutants. Lateral root growth deficiency was not significant in the absence of ABA (S9C Fig, *p* = 0.1, two-sample *t* test, Origin) but was observed in the *gef1/4/10/14* quadruple mutant plants compared with wild-type plants in the presence of ABA (S9D Fig, *p* = 0.02, two-sample *t* test, Origin).

## Discussion

RopGEFs were identified as plant-specific guanine nucleotide exchange factors [23,24]. GEF-ROP/RAC interactions and molecular mechanisms by which RopGEFs activate the small GTP-binding ROPs have been well established and clearly demonstrated [25,26]. In plants, ROPs are central regulators of many important cellular processes, including polar cell growth [57,58] and asymmetric cell division [59]. ROPs participate in ABA signaling [29,30,56]. However, how the abscisic acid signal controls the behavior of ROPs has remained unknown. In this study, we provide cytological, protein–protein interaction and genetic evidence to support that GEF1 protein abundance is controlled by ABA-mediated degradation, which would inactivate ROPs, releasing the inhibition of ABA signal transduction by ROPs.

### A Control Circuit in ABA Signal Transduction

Biochemical and structural studies have revealed the molecular mechanism by which ABA receptor proteins that have a higher probability of residing in a monomeric state constitutively bind to and down-regulate PP2Cs [9,10,60–62]. Eukaryotic receptor signal transduction often relies on regulators that ensure that signal transduction is shut off in the absence of a stimulus [11–13]. An earlier study showed that a small GTPase ROP11 can directly bind to ABI1 and release ABI1 from phosphatase activity inhibition by the monomeric ABA receptor RCAR1/PYL9 [31]. Here, we report that PP2Cs in turn protect RopGEF1 from ABA-mediated degradation. Considering the well-established activation relationship between RopGEFs and ROPs, we propose a RopGEF-ROP-PP2C control loop model that can effectively shut off ABA signal transduction in the absence of ABA (Fig 8C).

Under non-stress conditions, basal ABA concentrations in plant cells are low, and monomeric PYL/RCARs bind to PP2Cs in the absence of ABA but with a low affinity [9]. This offers ROPs the opportunity to interfere with the leaky repression of PP2C activity by monomeric PYL/RCARs at low basal ABA concentrations [9,10,63,64] through competitive binding of ROP11 to PP2Cs (Fig 8C). In the absence of ABA, PP2Cs are active and also protect RopGEFs from degradation and thus maintain ROPs in an active form (Figs 5 and S6). These interactions predict that RopGEF-ROP-PP2Cs form a positive feedback circuit to counteract leaky ABA signal transduction (Fig 8C).

Under stress conditions, the ABA content in plant cells increases, and ABA-bound PYL/RCARs bind to PP2Cs with a higher affinity [9,10], which would put ROPs at a competitive disadvantage. Furthermore, ABA signal transduction causes an ABA-mediated rapid degradation of RopGEFs (Figs 1, 3 and 5) leading to inactivation of ROPs [25,26]. As a result, in the presence of ABA, the RopGEF-ROP-PP2Cs circuit collapses rapidly, and PP2Cs are fully inactivated by ABA, releasing SnRK2 protein kinases to trigger ABA responses [1,3]. Genetic analyses of higher-order *pp2c*, *gef*, and *pyl/rcar* mutants correlate with this model (Figs 5–8 and S7–S9 Figs). Recent research revealed that ABI1 can interact with the U-box E3 ligases PUB12 and PUB13 and is subsequently degraded through the proteasome. However, PUB12/13 mediated ubiquitination of ABI1 only occurs when ABI1 interacts with PYL/RCAR ABA receptors [65]. This finding may imply that release of ABI1 from constitutive binding to monomeric PYL/RCARs, for example, through ROPs, would be essential to maintain ABI1 activity.

The RopGEF-ROP-PP2C circuit loop model exhibits several advantages. First, it could provide a mechanism for dynamic responses to ABA content changes in response to stress [2] and provide a reasonable explanation for binding affinities of PYL/RCARs to PP2Cs [9,10], and for the ability of ROPs to protect PP2C activity [31,56].

The proposed control loop may further provide a mechanism for establishing network linkage of ABA signal transduction with other physiological signals and critical cellular processes during plant growth. Physiological signals that affect activities of GEFs and ROPs will impact ABA signal transduction, especially considering that RopGEFs act as a bridge to link various extracellular signals [27,28,66,67]. On the other hand, ABA degradation of GEF1 can also contribute to the response of plants to adverse abiotic stress conditions via down-regulation of plant growth [68,69]. ROPs regulate a broad spectrum of cellular processes such as polarized cell growth, cell division, and cell wall restructuring [57,59,70]. Thus, ABA signal transduction may be able to exert a substantial influence on plant growth through the control of GEF-ROP activities, as shown here for the function of ABA signal transduction in GEF1 regulation of lateral root growth.

### RopGEFs Are Negative Regulators of ABA Signaling Transduction

Based on ABA-mediated particle formation among GEFs, we constructed *gef1/4/10 gef1/4/14* triple mutants, and the *gef1/4/10/14* quadruple mutant to reduce possible overlapping *GEF* gene functions. The quadruple mutant *gef1/4/10/14* shows enhanced ABA sensitivity in ABA-mediated inhibition of seed germination (Fig 7F, see also S11 Fig). Our findings do not rule out the possibility that additional mechanisms exist to regulate GEF activity by ABA signal transduction, as supported by a recent report that ABA causes degradation of RopGEF2 through the ubiquitin-26S proteasome system [71].

### RopGEF1 Plays an Important Role in ABA-Mediated Inhibition of Lateral Root Growth

Root branching architecture is a crucial determinant of nutrient and water uptake and lodging resistance of plants. The initiation and postemergence of lateral roots are coordinately regulated by the plant hormones auxin [72,73] and ABA [69,74], microRNAs [75], and environmental responses such as hydrotropism [76]. ABA treatment induces growth quiescence in lateral roots, whereas expression of *abi1-1*, which dominantly inhibits ABA signal transduction, leads to a recovery in lateral root growth [69]. In the present study, two *GFP-GEF1* overexpression lines, *GFP-GEF1/*WT and *GFP-GEF1/pyr1pyl124*, exhibited longer average lateral root lengths compared to their respective background lines, suggesting that GEF1 has the ability to facilitate lateral root growth (S8A Fig). More importantly, this ability of GFP-GEF1 is inhibited by application of ABA in wild-type but not in *pyr1/pyl1/2/4* plants (S8B Fig), implicating a contribution of ABA-mediated down-regulation of GEF1 function.

The *abi1/abi2/hab1/pp2ca* quadruple mutant plants exhibited a severe deficiency in lateral root growth (Figs 8A, 8B and S7E–S7G) and low protein abundance of GEF1 (Fig 5). Furthermore, overexpression of *GEF1* in *abi1/abi2/hab1/pp2ca* plants partially restored lateral root growth (Fig 8A and 8B), which may be attributed to the finding that GEF1 also interacts with additional clade A PP2Cs including HAI1 and AHG1 (S5B and S5C Fig). We speculate that ABA-mediated degradation of GEFs may play an important role in ABA-inhibition of lateral root growth.

### PP2C Protein Phosphatases Protect GEF1 from Degradation

It was previously shown that AtRAC1/ROP6 activity is rapidly inactivated by ABA in wild-type but not in *abi1-1* cells [29]. This could be explained by the present model (Fig 8C), as RopGEFs, the activators of ROPs, are rapidly degraded by ABA (Figs 1 and 3). GEF degradation leads to the inactivation of ROPs [23–25]. In the *abi1-1* mutant, the constitutively active ABI1 phosphatase [4,5,77,78] protects GEF1 from degradation, thus maintaining ROPs in an active form. This is also supported by experimental results showing that both ABA-mediated formation of intracellular GFP-GEF1 particles and degradation of GFP-GEF1 were substantially dampened by overexpression of mCherry-abi1-1 (S10 Fig). Furthermore, our results show that GEF1 directly interacts with ABI1 in Y2H, BiFC, and pull-down assays (Figs 4, S4 and S5A), and GEF1 undergoes constitutive degradation in *abi1/abi2/hab1/pp2ca* plants (Fig 5). Considering the importance of intact PP2Cs for inhibiting GFP-GEF1 degradation (Fig 5), we speculate that a potential protein kinase can phosphorylate RopGEF1 in response to PP2C inhibition by ABA. RopGEF1 phosphorylation [28] may subject RopGEF1 to degradation (Fig 8C). In the absence of ABA or at low basal ABA concentrations, PP2C-mediated dephosphorylation of RopGEF1 may protect RopGEF1 from degradation, which in turn can prevent leaky ABA signal transduction. Further research will be needed to determine whether the PP2Cs directly dephosphorylate RopGEF1 and to identify protein kinases that may function in signaling of RopGEF1 degradation, and also to determine whether ABA-activated protein kinases such as the SnRK2 protein kinases [79,80] are involved in ABA-induced RopGEF1 degradation.

## Materials and Methods

### Plant Material and Growth Conditions

All *Arabidopsis thaliana* lines were in the Columbia (Col-0) background. The *gef1* (SALK_058164C), *gef4* (CS808780), *gef10* (SALK_009456C), and *gef14* (CS820474) seeds and plasmids CD3-983, CD3-991, and CD3-959 were obtained from the *Arabidopsis* Biological Resource Center. Accession numbers for *gef1/4/10* and *gef1/4/10/14* mutants are CS69175 and CS69176. *Arabidopsis* seeds were surface sterilized in 20% bleach for 30 min followed by four washes with sterile water and sown on 1/2 MS media (pH 5.8) supplemented with 1% sucrose and 0.8% Phyto Agar. Plates with sterilized seeds were stratified in the dark for 3 d at 4°C and then transferred to the growth room. The growth conditions were as follows: 16/8 light/dark cycle, 80 μmol m^-2^s^-1^ light intensity, 22 to 24°C, and 30% relative humidity. 1-wk-old seedlings were transplanted into soil.

### Plasmid Construction and Plant Transformation

Coding sequences were amplified from mixed *Arabidopsis* flower, leaf, and seedling cDNA. Indicated fusion constructs were generated through USER technology [81]. After the sequences were verified, the resulting constructs were transformed into Agrobacterium strain GV3101. Transgenic lines were generated through the standard floral dip method.

### Transient Expression in *N*. *benthamiana* Leaves

Optical density (OD_600)_ of *Agrobacterium* cells was adjusted to 0.3 with buffer (10 mM MES, 10 mM MgCl_2_, 100 μM acetosyringone, pH 5.6). For co-expression, equal volumes of bacterial suspensions were mixed at a final OD_600_ of 0.3 each and infiltrated into 6-wk-old *N*. *benthamiana* leaves with a syringe and needle. 48 h after infiltration, the leaves were injected with 50 μM ABA or control buffer (0.1% v/v EtOH) and GEF1 fluorescence confocal imaging was conducted 1 h after injection or at the indicated times.

### Protoplast Preparation and Vacuole Release

GFP-GEF1 and mCherry-γ-TIP constructs were co-expressed in *N*. *benthamiana* leaves, and ABA was applied as described above. At 48 h after transfection, mesophyll protoplasts from *N*. *benthamiana* leaves were isolated by enzymatic digestion. The enzyme solution contained 1% (w/v) cellulase R-10, 0.25% (w/v) macerozyme, 10 mM CaCl_2_, 20 mM KCl, 400 mM mannitol, and 20 mM MES, pH 5.7. Protoplasts were washed twice by centrifugation at 80 g for 2 min and resuspended in the same solution without enzymes. Vacuoles were released through mixing protoplasts with lysis buffer (100 mM malic acid, 3 mM MgCl_2_, 0.1 mM CaCl_2_, pH adjusted to 7.5 with bis-Tris propane, and osmolarity adjusted to 500 mOsm with sorbitol).

### Confocal Microscopy

Fluorescence signals were detected using an LSM 710 confocal microscope (Zeiss) with a 20X objective lens. The following wavelengths were used for fluorescence detection: excitation 488 nm and emission 490–530 nm for GFP; excitation 488 nm and emission 520–550 for YFP; excitation 543 nm and emission 560–620 nm for mCherry and FM4-64; excitation 350 nm and emission 430–480 nm for DAPI. For co-localization analyses, GFP and mCherry fluorescence signals were acquired using the sequential line scanning mode to avoid bleed-through. Confocal imaging data were repeated at least in three independent experiments. For each condition and each experiment, at least five independent cells were analyzed in separate *N*. *benthamiana* leaves or *Arabidopsis* roots when indicated. Representative images are shown in the figures. In order to quantify co-localization results, the linear Pearson (rp) and the non-linear Spearman’s rank (rs) correlation coefficient (PSC) were calculated using FIJI software with an intensity correlation analysis plugin (http://www.uhnresearch.ca/facilities/wcif/software/Plugins/ICA.html). Levels of co-localization for representative areas (yellow boxes in figures) are depicted in intensity scatter plots. Calculated PSC values are given in the upper left corner of scatter plots.

For time-lapse observations, a rectangular well was made on slides with dow corning high vacuum grease and filled with an ABA-containing 1/2 MS solution. Roots of seedlings or *N*. *benthamiana* leaf discs were mounted in solution with a coverslip for confocal microscopy. For *Arabidopsis* leaves, young 2-wk-old plants were used and the abaxial epidermis was imaged in intact leaves. Single confocal focal planes and confocal images were recorded at the indicated times.

### Y2H Experiments

Y2H assays were performed with the USER-modified pGBT9 and pGADGH vectors (Clontech). Indicated bait and prey constructs were transformed into Yeast PJ69-4A cells and selected on SD-L-W medium. Yeast colonies were re-streaked onto new SD-L-W plates and incubated 1 or 2 d. Successfully transformed clones were incubated in SD-L-W liquid medium overnight and then the OD_600_ of cultures was adjusted to 1 with sterile water. A series of 2 μl 10-fold dilutions of transformants were spotted on SD-L-W and SD-L-W-H supplemented with 3 mM 3-amino-1,2,4-triazole (3-AT), and grown for 6 d.

### Pull-Down and Western Blot Analysis

The coding sequence of RopGEF1 was cloned into the pGEX6P-1 (GE Healthcare) to generate a GST-GEF1 fusion construct, and coding sequences of ABI1, OST1, and ROP11 were cloned into the modified pET52strep-II vector to generate Strep-II tag labeled ABI1, OST1, and ROP11 fusion constructs. After sequences were verified, the constructs were transformed into *E*. *coli* Rosetta (DE3) pLysS (Novagen) cells, and expression of the fusion protein was induced by 0.5 mM IPTG (isopropylthio-β-galactoside) at 18°C overnight (1 L overnight bacterial cultures were used for purification of StrepII-ABI1). GST and strep-II fusion proteins were purified using Gluotathione Sepharose^TM^ fast flow (GE healthcare, Pittsburgh, US) and *Strep***-**Tactin resin (IBA, Goettingen, Germany), respectively, according to the manufacturers’ instructions. Pull-down assays were performed according to [82]. Bound proteins were eluted, fractionated by 10% SDS-PAGE, and subjected to immune-blot analysis using *Strep*-Tactin HRP conjugate (IBA, Göttingen, Germany). For western blot assays, 10-d-old *Arabidopsis* seedlings grown on 1/2 MS medium were transferred to 1/2 MS liquid media for 1 h followed by hormone or chemical treatments for the indicated times. Seedlings were ground in liquid nitrogen and then resuspended in an extraction buffer containing 25 mM Tris-HCl (pH 7.5), 150 mM NaCl, 10% glycerol, 10 mM DTT, 1 mM EDTA, 0.5% Triton X-100, 1 mM PMSF, 10 mM NaF, and 1 × protease inhibitor cocktail (Roche) and incubated on ice for 30 min. Cell debris was pelleted by centrifugation at 15000 g for 20 min at 4°C; the supernatant was transferred to new tubes. Protein concentration was determined using the Bradford protein assay kit (IBI Scientific, Peosta, US). 20 μg proteins were separated on 10% SDS-PAGE gel, blotted onto a nitrocellulose membrane (Milli-pore), probed with anti-GFP antibody (Life Technology) overnight at 4°C, and then incubated with a goat anti-rabbit HRP-conjugated secondary antibody (Bio-Rad) for 1 h at room temperature. Membranes were incubated with chemiluminescence reaction solution (Pierce, Rockford, US) and western blot signal was detected using a typhoon Imager FLA 7000 (GE healthcare, Pittsburgh, US).

### Co-IP Analyses

*Agrobacterium* cells containing RK19 (to reduce the silencing of transgenes) were mixed with those containing *pUBQ*-*Flag-GFP-GEF1* or *pUBQ*-*myc-mCherry-PP2C* constructs at a ratio of 1:1. Subsequently, the mixture was co-injected into *N*. *benthamiana* leaves. At 48 h after infiltration, *N*. *benthamiana* leaves were ground in liquid nitrogen and homogenized in protein extraction buffer (25 mM Tris-HCl, pH 7.5, 150 mM NaCl, 10% glycerol, 0.1% Nonidet P-40, 1 mM EDTA, 10 mM DTT, 1 mM phenylmethylsulfonyl fluoride, and 1 × complete cocktail of protease inhibitors, 2 ml buffer/g leaves). Lysates were vigorously votexed for 30 s, then incubated in ice for 30 min. After centrifugation at maximal speed 15000 g for 15 min at 4°C, the supernatant was transferred into a new tube and passed through a 0.45 μm filter (Millipore). 30 μl of flag magnetic beads (Sigma) were added to 5 mg of total proteins, and the mixture was incubated for 4 h at 4°C. The precipitated samples were washed four times with the protein extraction buffer (rotated at 4° C for 5 min, beads were collected with DynaMag^TM^-2 [Life Technology]) and then eluted by boiling in 2 × SDS loading buffer for 5 min. Immunoprecipitation products were detected by immunoblotting with rabbit monoclonal flag (Sigma) or mouse monoclonal myc antibody (Life Technology).

### Quantitative Reverse Transcription PCR Analyses

Fifteen 10-d-old seedlings were collected into 2 ml tubes with steel beads, frozen in liquid nitrogen, and ground with a Mixer Mill MM400 (Retsch). Total RNA was extracted using the SpectrumTM Plant Total RNA kit (Sigma) and quantified. Approximately 3 μg RNA samples were treated with 1 μl DNase I (NEB) for 30 min and converted to cDNA using a First-Strand cDNA Synthesis kit (GE Healthcare). Synthesized cDNA was diluted four times and 2 μl was used as PCR templates. qPCR analyses were performed on a plate-based BioRad CFX96 qPCR System using SYBR Select Master Mix for CFX (Applied Biosystems) with gene-specific primers. GEF1fwd: tgcttgccgaaatggagattccc; GEF1rev: agacattccttcccgctcttgg; GAPCfwd: tcagactcgagaaagctgctac; GAPCrev: cgaagtcagttgagacaacatcatc.

### Seed Germination and Root Elongation Assays

After surface sterilization of the seeds, 70 seeds of each genotype were sowed on 1/2 MS plates supplemented with ABA. Stratification was conducted in the dark at 4°C for 3 d. Radical emergence was recorded at the indicated times. At 6 d, seedlings with expanded green cotyledons were scored as the percentage of seeds. For root elongation assays, seeds were stratified for 3 d in the dark at 4°C (for experiments including *abi1abi2hab1pp2ca* quadruple mutant, all seeds were stratified for 10 d), sown, and grown on vertically oriented 1/2 MS plates for 4 d. Twenty seedlings with similar primary root length were transferred onto new 1/2 MS plates lacking or supplemented with the indicated concentrations of ABA. The plates were scanned after 6 d of growth, and primary root lengths were measured with ImageJ (http://imagej.nih.gov/ij/download.html).

### Lateral Root Growth Assays

Twenty 4-d-old seedlings grown on 1/2 MS medium with similar primary root lengths were transferred onto new 1/2 MS plates lacking or supplemented with the indicated concentrations of ABA and IAA. The plates were scanned after the indicated additional times of growth, and primary root lengths and total lateral root lengths (lateral roots longer than 0.3 mm were measured and counted) were measured with ImageJ. Average lateral root length was defined as total lateral root length divided by lateral root number for each root. Lateral root (LR) number/centimeter are lateral root number divided by primary root length. Because different hormones have different effects on lateral root growth, it is difficult to compare the effect of different hormone treatments at the same time. For example, in the present study, lateral roots were too dense to be distinguished on auxin or auxin plus ABA medium plates when lateral roots were imaged >4 d after transfer of seedlings, and the visible lateral roots on MS medium have just emerged. Therefore, plates were scanned and lateral roots were measured at the indicated times when lateral root lengths could be accurately measured. Comparisons were made among genotypes within each group with identical hormone treatments.

All relevant data are within the paper and its Supporting Information files.

## Supporting Information

S1 Data Underlying data for Figs 6A, 6B, 6D, 6F, 7A, 7B, 7D, 8, S4, S7, S8A, S8B, S8C, and S9. (XLSX)

S1 Fig Subcellular localization analysis of GFP-GEF1 in *N*. *benthamiana* leaves in the absence or presence of added ABA. GEF1 is localized in the cell periphery and cytosol in the absence of ABA (A, B, C), shows punctate fluorescence in the cytosol, and also moves into the nucleus in the presence of ABA (D). Fusion of GFP to the C- or N-termini of GEF1 does not affect the subcellular localization as shown in (A) and (B), respectively. Yellow arrow in (C) points to cell membrane after plasmolysis with 0.8 M NaCl for 5 min. Yellow asterisks in (D) point to punctate fluorescence in the cytosol, and white arrows point to nuclei. DAPI: (4',6-diamidino-2-phenylindole, stains nuclei). *N*. *benthamiana* leaves were treated with 50 μM ABA for 1 h, or 1 μg/ml DAPI for 10 min before confocal imaging. Scale bars: 10 μm. (E) Co-localization analyses of GFP-GEF1 with cell membrane dye FM4-64 in the absence (top panels) or the presence (bottom panels) of ABA. *N*. *benthamiana* leaves were treated with 50 μM ABA for 1 h, or 10 μM FM4-64 for 10 min before confocal imaging. Scale bars: 10 μm. (F) Root hair phenotypes in 4-d-old seedlings of wild-type, *35S-ROP11CA*, *35S-GEF1*, and *pUBQ-GFP-GEF1* transgenic plants. Confocal imaging experiments were repeated at least 3 times, with >5 cells analyzed per experiment. Scale bars: 100 μm.
(TIF)

S2 Fig Subcellular localization of GFP-GEF1 in response to the indicated hormone treatments. (A) Effects of the indicated hormone treatments on the subcellular localization of GFP-GEF1 at the indicated time points. (B) Subcellular localization of GFP-GEF1 in response to ABA or ACC treatment in *pyr1pyl124* ABA receptor quadruple mutant. Seven-day-old *Arabidopsis* seedlings overexpressing GFP-GEF1 in the *pyr1pyl124* quadruple mutant were treated with the indicated concentration of ABA or ACC or control EtOH for 3 h before confocal imaging. ABA at 100 μM only partially caused particle formation after 3 hours in the *pyr1pyl124* quadruple mutant compared to WT (A). (C) Identification of *proGEF1-GFP-GEF1* transgenic line by PCR. Genomic DNA was extracted for PCR reactions. Black arrows indicate binding sites of PCR primers. (D) Subcellular localization of GFP-GEF1 driven by the *RopGEF1* promoter (1,983 bp sequence containing the 5’UTR region of GEF1) in *Arabidopsis* leaf epidermes in response to ABA or 0.1% (v/v) EtOH treatments. (E) Subcellular localization of GFP-GEF1 in control *N*. *benthamiana* leaves without ABA addition at the indicated time points of confocal analyses. Scale bars 10 μm.
(TIF)

S3 Fig Subcellular localization analyses of GFP-GEF1 in response to ABA. (A, B) Co-localization analysis of GFP-GEF1 with ER, *cis*-Golgi, late endosome markers (A) and lytic vacuolar marker γ-TIP and sheet-like ER (B) in *N*. *benthamiana* leaves. GFP-GEF1 and mCherry-labeled organelle markers were co-expressed in *N*. *benthamiana* leaves. At 48 h after infiltration, *N*. *benthamiana* leaves were treated with 50 μM ABA for 1 h before confocal imaging. Organelle marker names are listed in parentheses. Representative images are shown of co-localization experiments. Yellow boxes indicate approximate regions used for correlation analyses. Images to the right of merged images depict parts of boxed fields. Levels of co-localization for yellow boxed regions are depicted in relative intensity (*x-* and *y*-axes) scatter plots. Values of the linear Pearson correlation coefficient (rp) and the non-linear Spearman’s rank correlation coefficient (rs) were calculated and are given in the upper left corner of scatter plots. Scale bars 10 μm. (C) The effect of Brefieldin A (BFA) on subcellular localization of GFP-GEF1 in roots of *GFP-GEF1* overexpression lines in response to ABA treatment. Four-day-old *GFP-GEF1*/WT overexpression seedlings were treated with 50 μM ABA plus 50 μM BFA for 1 h before confocal imaging. ABA plus 0.1% (v/v) DMSO treatment was used as a control. Yellow arrows point to BFA bodies.
(TIF)

S4 Fig **BiFC assays of interactions of GEF1 with the indicated PP2Cs in *N*. *benthamiana* leaves (A) and quantification of relative fluorescence intensities (relative to that of ROP11-GEF1 interaction) in BiFC analyses (B).** YFP^N^/YFP^C^-GEF1 and YFP^C^/YFP^N^-ABI1/ABI2/HAB1/PP2CA were used as negative controls. Data represent mean ± SD of three independent replicates. Ten cells were analyzed in each replicate for each construct combination. Scale bars: 10 μm. Images were acquired using identical settings, Zeiss LSM 710 (objective: 20x; laser: 488; filter: 520–550; pinhole: 90 μm; digital gain: 1; channel: 8 bit; average: line 4; zoom: 1; master gain: 800).
(TIF)

S5 Fig RopGEF1 interacts with PP2C phosphatases. (A) Subcellular localization of mCherry-ABI1 and co-localization of GFP-GEF1 and mCherry-ABI1. Scale bars 10 μm. (B) Y2H assay of interactions of GEF1 with the indicated PP2C phosphatases. The indicated construct combinations were co-transformed into the yeast strain pJ69-4A. Transformants were grown on -L-W control plates (left) for 3 d and -L-W-H (lacking Leucine, Tryptophan, and Histidine) selective plates with 5 mM 3-amino-1,2,4-triazole (3-AT) (right) for 6 d. (C) Co-immunoprecipitation (Co-IP) assay of interactions of GEF1 with the indicated PP2C phosphatases in *N*. *benthamiana* leaves. Co-IP was carried out with anti-flag magnetic beads, and immunoblotting analyses were performed with anti-flag and anti-myc antibody. Input, total protein extracts for immunoprecipitates; IP, immunoprecipitates; molecular weight markers (in kD) are shown on the right.
(TIF)

S6 Fig Effect of Wortmannin treatment on cell localization and protein level of GFP-GEF1 in *GFP-GEF1*/ *abi1abi2hab1pp2ca* overexpression lines. (A) The effect of Wortmannin on the subcellular localization of GFP-GEF1 in *abi1/abi2/hab1/pp2c* plants. Four-day-old seedlings grown on 1/2 MS medium were treated with 20 μM Wortmannin for 1 h (A) and 3 h (B). (C) Subcellular localization of GFP-GEF1 in root cells in the differentiation zone of a primary root and a newly emerging lateral root tip (top). GFP-GEF1 gathers into a round mass in newly emerging lateral root tip cells in which functional lytic vacuoles have been reported to not yet have developed (see Results) [55]. White asterisks show ring-like structures induced by Wortmannin treatment. Yellow box indicates a magnified ring-like structure. White arrows point to round structures surrounding nuclei as shown for the subcellular localization of GFP-GEF1 in the wild-type background (S1A–S1C Fig). Yellow arrow points to GFP-GEF1 punctate structure in cells. Images in (A–C) were acquired using identical confocal parameters, and image brightness was identically adjusted with ImageJ software to enhance visibility of the weak fluorescence signal in *GFP-GEF1*/ *abi1abi2hab1pp2ca* seedlings. (D, E) Immuno-blotting analyses of GFP-GEF1 protein levels in GFP-GEF1 overexpression lines in *abi1abi2hab1pp2ca* (D) and wild-type (E) background after Wortmannin treatment. Ten-day-old *Arabidopsis* seedlings were treated with 20 μM Wortmannin or 0.1% (v/v) DMSO for 3 h. Total protein was extracted and immune-blots were carried out with GFP antibody.
(TIF)

S7 Fig Overexpression of *GFP-GEF1* in *abi1/abi2/hab1/pp2ca* plants partially rescues lateral root deficiency of quadruple mutant. Quantification of average lateral root number in wild-type, *abi1-1*, *abi1/abi2/hab1/pp2ca*, and *pUBQ-GFP-GEF1*/*abi1abi2hab1pp2ca* overexpression plants. Lateral root (LR) number/centimeter are defined as visible lateral root (>0.3 mm length) number relative to primary root length. Data are mean ± SD of three independent replicates. Twenty seedlings per replicate and condition. Note that the bar graphs in A–D are from different time points to accurately resolve lateral root lengths for each condition. Only lateral roots lengths >0.3 mm length were counted (See Methods). (E–G) Representative images of lateral root growth of different seedling genotypes grown on 1/2 MS medium (E) or supplemented with 10 μM ABA (F) or 0.1 μM IAA plus 10 μM ABA (G). Four-day-old seedlings grown on 1/2 MS medium were transferred onto 1/2 MS medium or supplemented with 10 μM ABA, 0.1 μM IAA, or both. After the indicated times of growth (5 d [E], 8 d [F], and 3 d [G]), images were taken and lateral roots longer than 0.3 mm were counted and measured. IAA was added to stimulate lateral root growth for enhanced visualization in (G).
(TIF)

S8 Fig RopGEF1 has ability to promote lateral root growth. (A and B) Quantification of average lateral root length in WT, *pUBQ-GFP-GEF1/*WT, *pyr1/pyl1/2/4*, and *pUBQ-GFP-GEF1/pyr1pyl124* overexpression plants. Data are mean ± SD of three independent replicates. Twenty seedlings analyzed per replicate and condition. *P* values were determined by two-sample *t* test, Origin; (**) *p* < 0.01; (*) *p* < 0.05; (*ns*) not significant. (C) Real-time quantitative PCR analyses of *RopGEF1* expression levels in seedlings of the indicated genotypes. Total RNAs were extracted from 10-d-old seedlings grown on 1/2 MS medium. The *GAPC* (glyceraldehyde-3-phosphate dehydrogenase C subunit) gene was used as an internal standard. Relative expression to wild-type Col is presented. Error bars are SD of three biological replicates.
(TIF)

S9 Fig Lateral root growth in higher order of *gef* mutants. (A) Identification of homozygous *gef1/4/10/14* quadruple mutant. LP and RP, gene-specific primers; LB, T-DNA specific primer; qua: *gef1/4/10/14* quadruple mutant. Genotyping primers were from http://signal.salk.edu/tdnaprimers.2.html. (B) RT-PCR analysis of expression levels of *GEF1*, *GEF4*, *GEF10*, and *GEF14* in wild-type and *gef1/4/10/14* quadruple mutant plants. PCR cycles were 20 for *ACTIN2* and 32 for *GEF*1, *GEF4*, *GEF10*, and *GEF14*. Full-length coding sequences of *GEF1*, *GEF4*, *GEF10*, and *GEF14* were amplified with 5′ and 3′ end primers. (C,D) Representative images of lateral root growth of indicated *gef* triple and quadruple mutant seedling genotypes grown on 1/2 MS medium supplemented with 0.1 μM IAA or 0.1 μM IAA plus 10 μM ABA. Note the reduced number of visible lateral roots in *gef1/4/10/14* quadruple mutant seedlings (far right in D) compared to wild-type (WT) seedlings (far left in D). (*) *p* < 0.05; (*ns*) not significant.
(TIF)

S10 Fig Subcellular localization and protein level of GFP-GEF1 in *Arabidopsis* expressing both GFP-GEF1 and mCherry-*abi1-1*. (A) Subcellular localization of GFP-GEF1 in response to ABA in *Arabidopsis* expressing both GFP-GEF1 and mCherry-*abi1-1*. Time after ABA treatment is indicated in the Merge images. (B) Immuno-blotting analyses of GFP-GEF1 protein levels in expression lines of both GFP-GEF1 and mCherry-*abi1-1*. Ten-day-old *Arabidopsis* seedlings were treated with 50 μM ABA for the indicated times. Total protein was extracted and immune-blotting was carried out with GFP antibody. Coomassie blue staining of SDS-polyacrylamide gel (SDS-PAGE) was used as a loading control.
(TIF)

S11 Fig Seed germination assay of the indicated *gef* double, triple and quadruple mutants, and controls of wild-type and *pyr1pyl124* quadruple mutant plants in 1/2 MS media with or without 0.5 μM ABA. Images of germinating seeds grown on 1/2 MS medium for 6 d (top) or on 1/2 MS media supplemented with 0.5 μM ABA for 3 d (bottom). The depicted plates are the same plate as magnified images shown in Fig 7F.
(TIF)

## Abbreviations

ABA: abscisic acid
BFA: Brefeldin A
BiFC: bimolecular fluorescence complementation
FLS2: FLAGELLIN SENSITIVE2
LR: lateral root
PI3K: phosphatidylphosphate-3-kinase
PP2C: type 2C protein phosphatase
PRONE: plant-specific ROP nucleotide exchanger
PVC: prevacuolar compartment
RLK: receptor-like kinase
Y2H: yeast-two-hybrid
