# Supplementary figures and images for: Release of GTP Exchange Factor Mediated Down-Regulation of Abscisic Acid Signal Transduction through ABA-Induced Rapid Degradation of RopGEFs
Source: PLoS Biol. 2016 May 18;14(5):e1002461. doi: 10.1371/journal.pbio.1002461 (PMC4871701; doi:10.1371/journal.pbio.1002461)

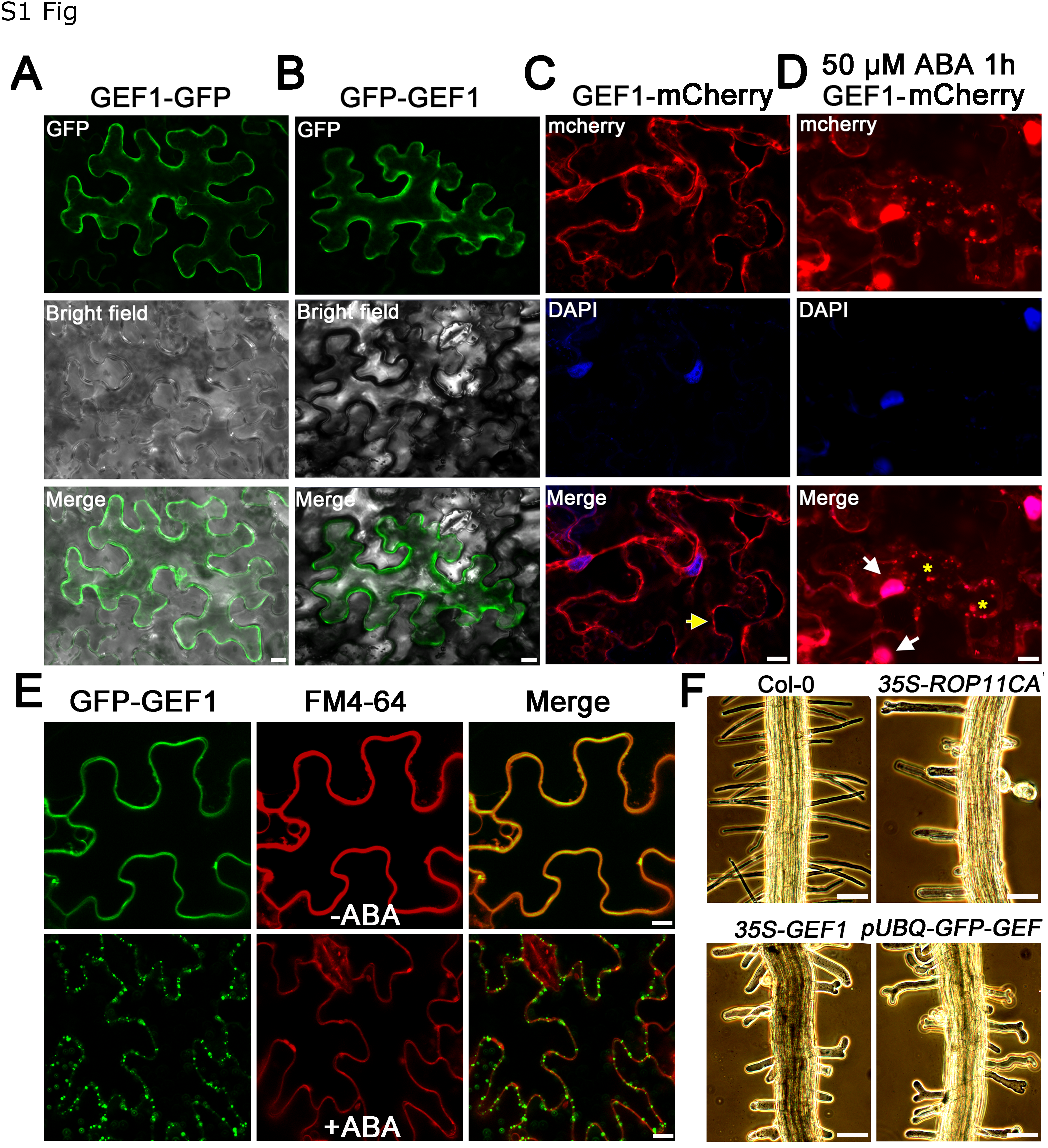

Supplement: S1 Fig — GEF1 is localized in the cell periphery and cytosol in the absence of ABA (A, B, C), shows punctate fluorescence in the cytosol, and also moves into the nucleus in the presence of ABA (D). Fusion of GFP to the C- or N-termini of GEF1 does not affect the subcellular localization as shown in (A) and (B), respectively. Yellow arrow in (C) points to cell membrane after plasmolysis with 0.8 M NaCl for 5 min. Yellow asterisks in (D) point to punctate fluorescence in the cytosol, and white arrows point to nuclei. DAPI: (4',6-diamidino-2-phenylindole, stains nuclei). N. benthamiana leaves were treated with 50 μM ABA for 1 h, or 1 μg/ml DAPI for 10 min before confocal imaging. Scale bars: 10 μm. (E) Co-localization analyses of GFP-GEF1 with cell membrane dye FM4-64 in the absence (top panels) or the presence (bottom panels) of ABA. N. benthamiana leaves were treated with 50 μM ABA for 1 h, or 10 μM FM4-64 for 10 min before confocal imaging. Scale bars: 10 μm. (F) Root hair phenotypes in 4-d-old seedlings of wild-type, 35S-ROP11CA, 35S-GEF1, and pUBQ-GFP-GEF1 transgenic plants. Confocal imaging experiments were repeated at least 3 times, with >5 cells analyzed per experiment. Scale bars: 100 μm. (TIF) [file pbio.1002461.s002.tif]

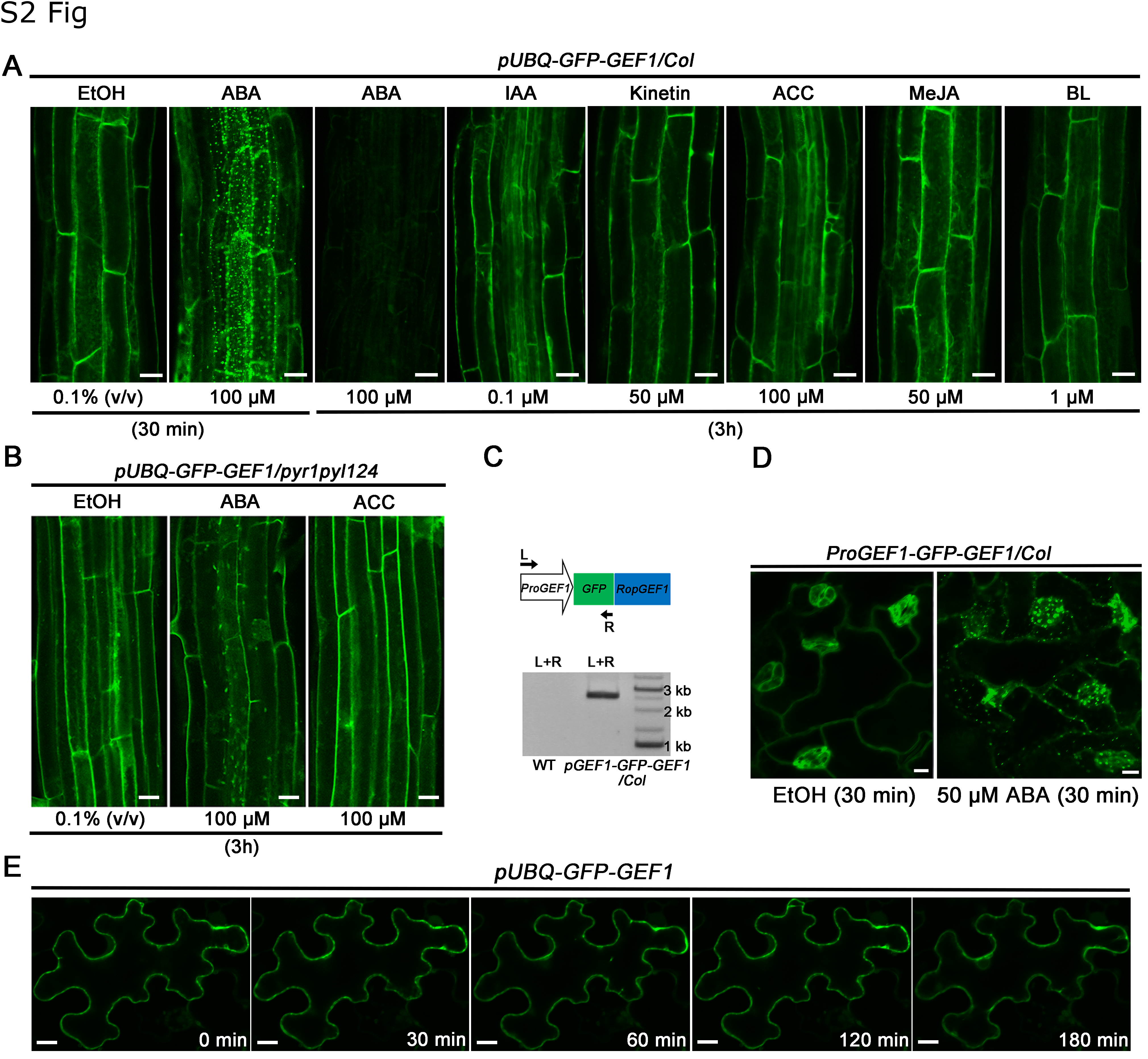

Supplement: S2 Fig — (A) Effects of the indicated hormone treatments on the subcellular localization of GFP-GEF1 at the indicated time points. (B) Subcellular localization of GFP-GEF1 in response to ABA or ACC treatment in pyr1pyl124 ABA receptor quadruple mutant. Seven-day-old Arabidopsis seedlings overexpressing GFP-GEF1 in the pyr1pyl124 quadruple mutant were treated with the indicated concentration of ABA or ACC or control EtOH for 3 h before confocal imaging. ABA at 100 μM only partially caused particle formation after 3 hours in the pyr1pyl124 quadruple mutant compared to WT (A). (C) Identification of proGEF1-GFP-GEF1 transgenic line by PCR. Genomic DNA was extracted for PCR reactions. Black arrows indicate binding sites of PCR primers. (D) Subcellular localization of GFP-GEF1 driven by the RopGEF1 promoter (1,983 bp sequence containing the 5’UTR region of GEF1) in Arabidopsis leaf epidermes in response to ABA or 0.1% (v/v) EtOH treatments. (E) Subcellular localization of GFP-GEF1 in control N. benthamiana leaves without ABA addition at the indicated time points of confocal analyses. Scale bars 10 μm. (TIF) [file pbio.1002461.s003.tif]

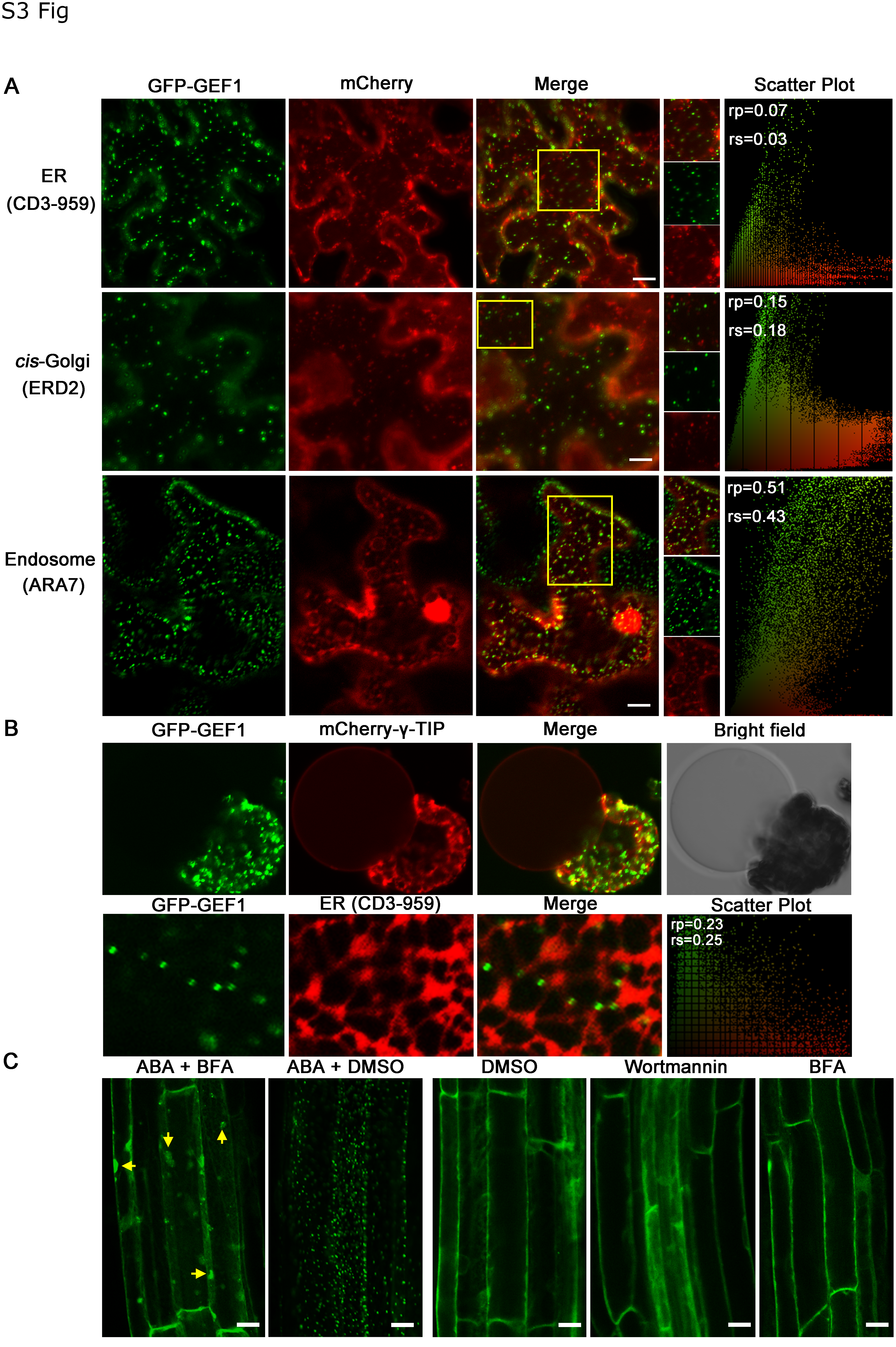

Supplement: S3 Fig — (A, B) Co-localization analysis of GFP-GEF1 with ER, cis-Golgi, late endosome markers (A) and lytic vacuolar marker γ-TIP and sheet-like ER (B) in N. benthamiana leaves. GFP-GEF1 and mCherry-labeled organelle markers were co-expressed in N. benthamiana leaves. At 48 h after infiltration, N. benthamiana leaves were treated with 50 μM ABA for 1 h before confocal imaging. Organelle marker names are listed in parentheses. Representative images are shown of co-localization experiments. Yellow boxes indicate approximate regions used for correlation analyses. Images to the right of merged images depict parts of boxed fields. Levels of co-localization for yellow boxed regions are depicted in relative intensity (x- and y-axes) scatter plots. Values of the linear Pearson correlation coefficient (rp) and the non-linear Spearman’s rank correlation coefficient (rs) were calculated and are given in the upper left corner of scatter plots. Scale bars 10 μm. (C) The effect of Brefieldin A (BFA) on subcellular localization of GFP-GEF1 in roots of GFP-GEF1 overexpression lines in response to ABA treatment. Four-day-old GFP-GEF1/WT overexpression seedlings were treated with 50 μM ABA plus 50 μM BFA for 1 h before confocal imaging. ABA plus 0.1% (v/v) DMSO treatment was used as a control. Yellow arrows point to BFA bodies. (TIF) [file pbio.1002461.s004.tif]

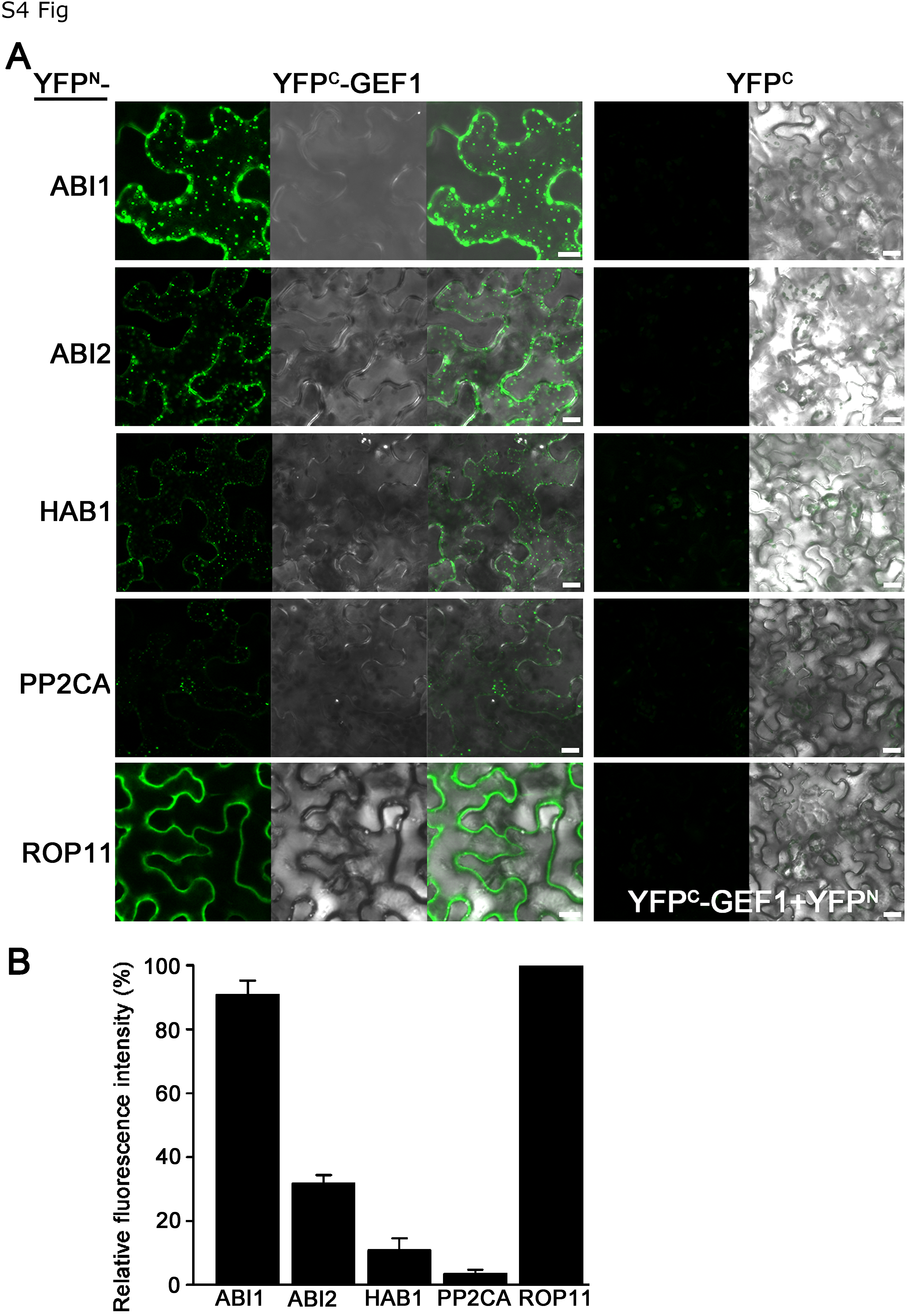

Supplement: S4 Fig — BiFC assays of interactions of GEF1 with the indicated PP2Cs in N. benthamiana leaves (A) and quantification of relative fluorescence intensities (relative to that of ROP11-GEF1 interaction) in BiFC analyses (B). YFPN/YFPC-GEF1 and YFPC/YFPN-ABI1/ABI2/HAB1/PP2CA were used as negative controls. Data represent mean ± SD of three independent replicates. Ten cells were analyzed in each replicate for each construct combination. Scale bars: 10 μm. Images were acquired using identical settings, Zeiss LSM 710 (objective: 20x; laser: 488; filter: 520–550; pinhole: 90 μm; digital gain: 1; channel: 8 bit; average: line 4; zoom: 1; master gain: 800). (TIF) [file pbio.1002461.s005.tif]

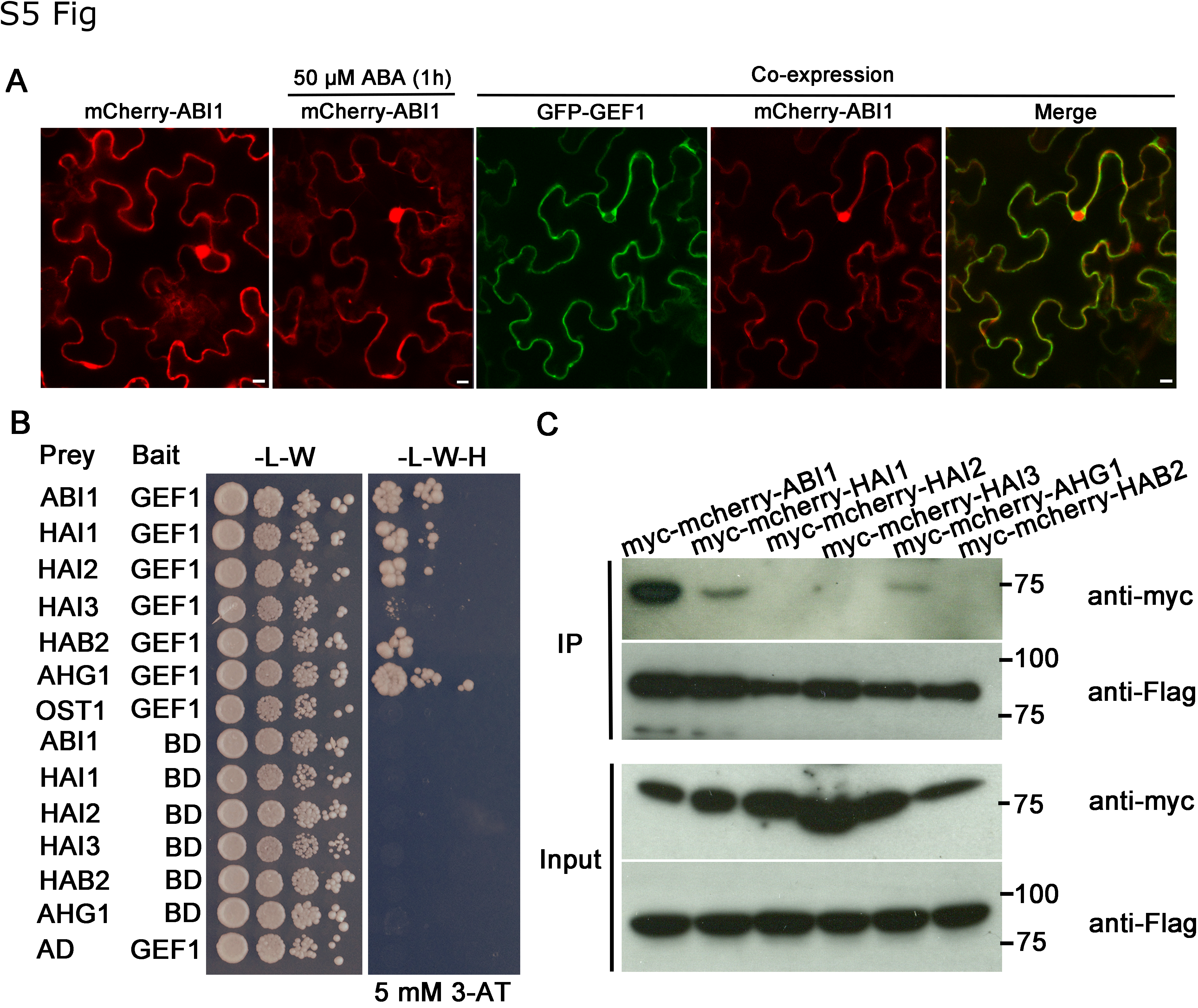

Supplement: S5 Fig — (A) Subcellular localization of mCherry-ABI1 and co-localization of GFP-GEF1 and mCherry-ABI1. Scale bars 10 μm. (B) Y2H assay of interactions of GEF1 with the indicated PP2C phosphatases. The indicated construct combinations were co-transformed into the yeast strain pJ69-4A. Transformants were grown on -L-W control plates (left) for 3 d and -L-W-H (lacking Leucine, Tryptophan, and Histidine) selective plates with 5 mM 3-amino-1,2,4-triazole (3-AT) (right) for 6 d. (C) Co-immunoprecipitation (Co-IP) assay of interactions of GEF1 with the indicated PP2C phosphatases in N. benthamiana leaves. Co-IP was carried out with anti-flag magnetic beads, and immunoblotting analyses were performed with anti-flag and anti-myc antibody. Input, total protein extracts for immunoprecipitates; IP, immunoprecipitates; molecular weight markers (in kD) are shown on the right. (TIF) [file pbio.1002461.s006.tif]

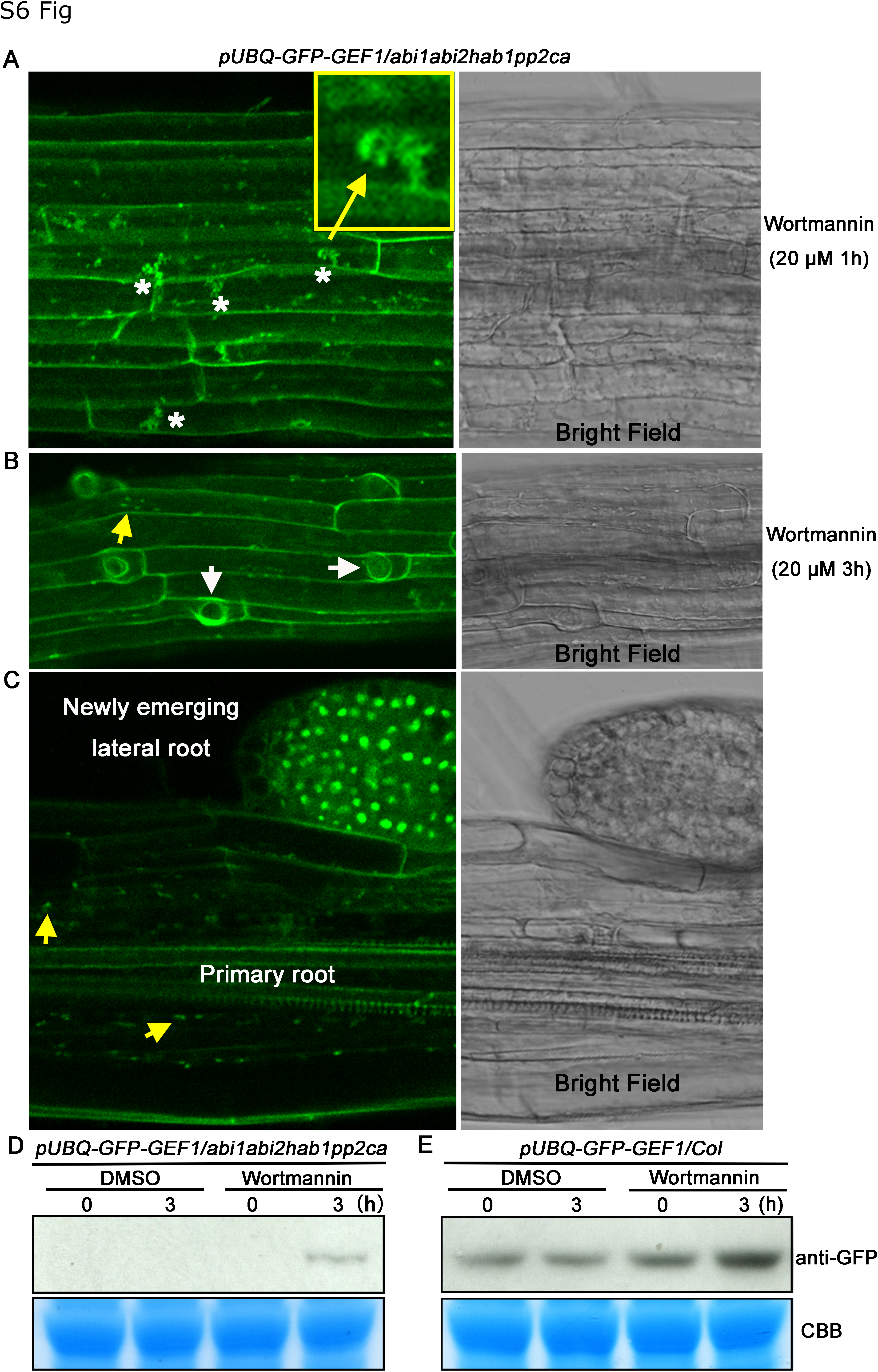

Supplement: S6 Fig — (A) The effect of Wortmannin on the subcellular localization of GFP-GEF1 in abi1/abi2/hab1/pp2c plants. Four-day-old seedlings grown on 1/2 MS medium were treated with 20 μM Wortmannin for 1 h (A) and 3 h (B). (C) Subcellular localization of GFP-GEF1 in root cells in the differentiation zone of a primary root and a newly emerging lateral root tip (top). GFP-GEF1 gathers into a round mass in newly emerging lateral root tip cells in which functional lytic vacuoles have been reported to not yet have developed (see Results) [55]. White asterisks show ring-like structures induced by Wortmannin treatment. Yellow box indicates a magnified ring-like structure. White arrows point to round structures surrounding nuclei as shown for the subcellular localization of GFP-GEF1 in the wild-type background (S1A–S1C Fig). Yellow arrow points to GFP-GEF1 punctate structure in cells. Images in (A–C) were acquired using identical confocal parameters, and image brightness was identically adjusted with ImageJ software to enhance visibility of the weak fluorescence signal in GFP-GEF1/ abi1abi2hab1pp2ca seedlings. (D, E) Immuno-blotting analyses of GFP-GEF1 protein levels in GFP-GEF1 overexpression lines in abi1abi2hab1pp2ca (D) and wild-type (E) background after Wortmannin treatment. Ten-day-old Arabidopsis seedlings were treated with 20 μM Wortmannin or 0.1% (v/v) DMSO for 3 h. Total protein was extracted and immune-blots were carried out with GFP antibody. (TIF) [file pbio.1002461.s007.tif]

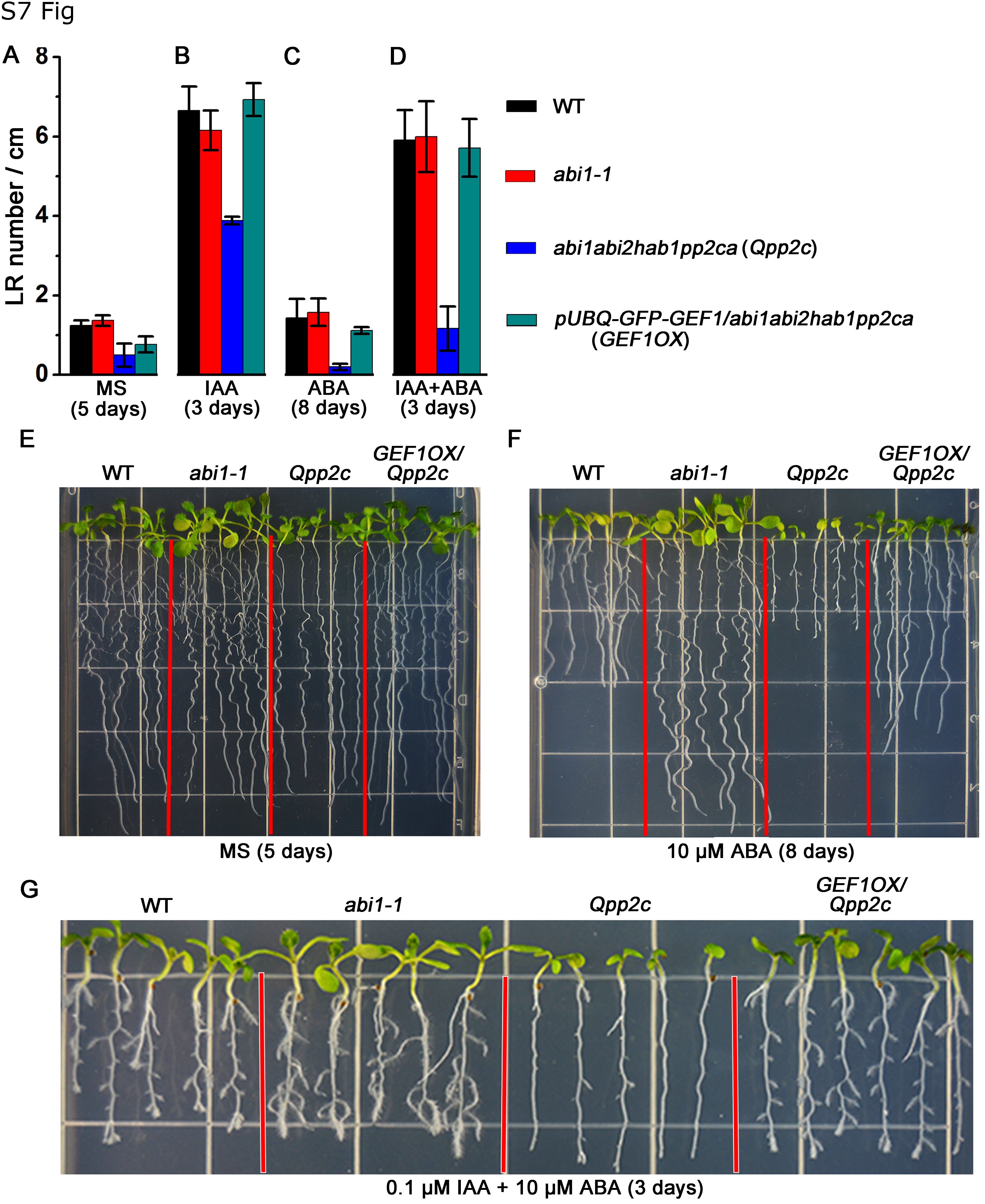

Supplement: S7 Fig — Quantification of average lateral root number in wild-type, abi1-1, abi1/abi2/hab1/pp2ca, and pUBQ-GFP-GEF1/abi1abi2hab1pp2ca overexpression plants. Lateral root (LR) number/centimeter are defined as visible lateral root (>0.3 mm length) number relative to primary root length. Data are mean ± SD of three independent replicates. Twenty seedlings per replicate and condition. Note that the bar graphs in A–D are from different time points to accurately resolve lateral root lengths for each condition. Only lateral roots lengths >0.3 mm length were counted (See Methods). (E–G) Representative images of lateral root growth of different seedling genotypes grown on 1/2 MS medium (E) or supplemented with 10 μM ABA (F) or 0.1 μM IAA plus 10 μM ABA (G). Four-day-old seedlings grown on 1/2 MS medium were transferred onto 1/2 MS medium or supplemented with 10 μM ABA, 0.1 μM IAA, or both. After the indicated times of growth (5 d [E], 8 d [F], and 3 d [G]), images were taken and lateral roots longer than 0.3 mm were counted and measured. IAA was added to stimulate lateral root growth for enhanced visualization in (G). (TIF) [file pbio.1002461.s008.tif]

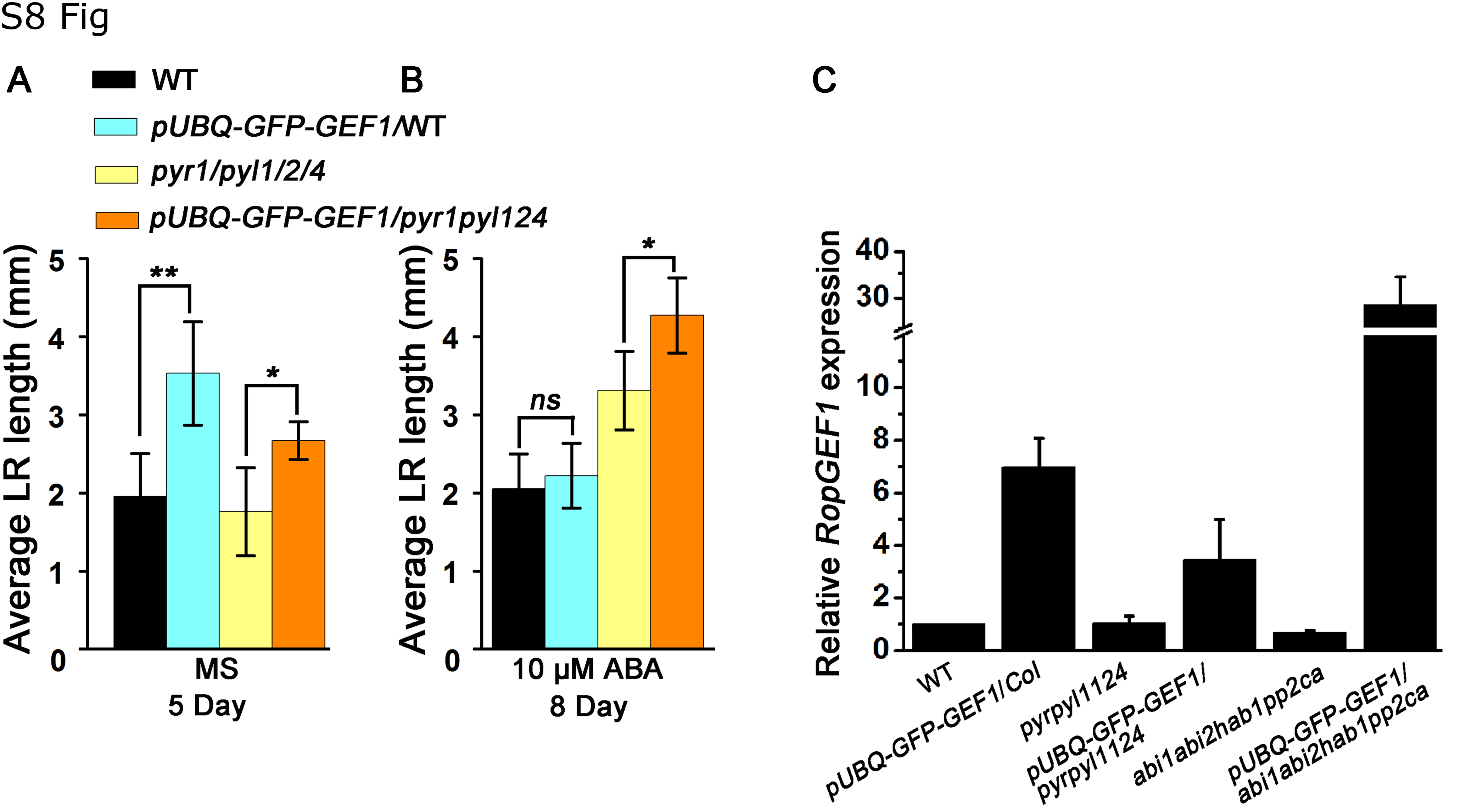

Supplement: S8 Fig — (A and B) Quantification of average lateral root length in WT, pUBQ-GFP-GEF1/WT, pyr1/pyl1/2/4, and pUBQ-GFP-GEF1/pyr1pyl124 overexpression plants. Data are mean ± SD of three independent replicates. Twenty seedlings analyzed per replicate and condition. P values were determined by two-sample t test, Origin; (**) p < 0.01; (*) p < 0.05; (ns) not significant. (C) Real-time quantitative PCR analyses of RopGEF1 expression levels in seedlings of the indicated genotypes. Total RNAs were extracted from 10-d-old seedlings grown on 1/2 MS medium. The GAPC (glyceraldehyde-3-phosphate dehydrogenase C subunit) gene was used as an internal standard. Relative expression to wild-type Col is presented. Error bars are SD of three biological replicates. (TIF) [file pbio.1002461.s009.tif]

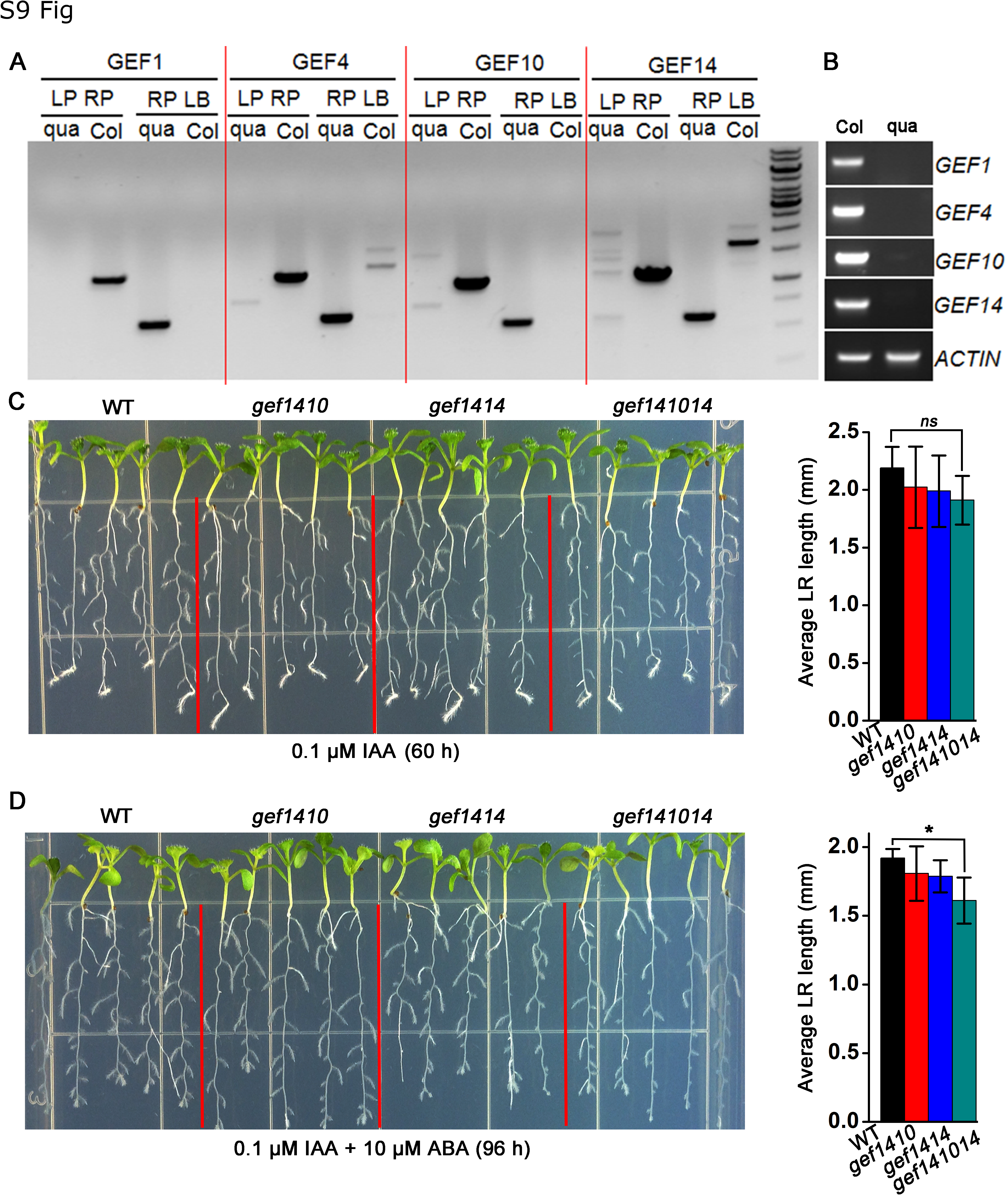

Supplement: S9 Fig — (A) Identification of homozygous gef1/4/10/14 quadruple mutant. LP and RP, gene-specific primers; LB, T-DNA specific primer; qua: gef1/4/10/14 quadruple mutant. Genotyping primers were from http://signal.salk.edu/tdnaprimers.2.html. (B) RT-PCR analysis of expression levels of GEF1, GEF4, GEF10, and GEF14 in wild-type and gef1/4/10/14 quadruple mutant plants. PCR cycles were 20 for ACTIN2 and 32 for GEF1, GEF4, GEF10, and GEF14. Full-length coding sequences of GEF1, GEF4, GEF10, and GEF14 were amplified with 5′ and 3′ end primers. (C,D) Representative images of lateral root growth of indicated gef triple and quadruple mutant seedling genotypes grown on 1/2 MS medium supplemented with 0.1 μM IAA or 0.1 μM IAA plus 10 μM ABA. Note the reduced number of visible lateral roots in gef1/4/10/14 quadruple mutant seedlings (far right in D) compared to wild-type (WT) seedlings (far left in D). (*) p < 0.05; (ns) not significant. (TIF) [file pbio.1002461.s010.tif]

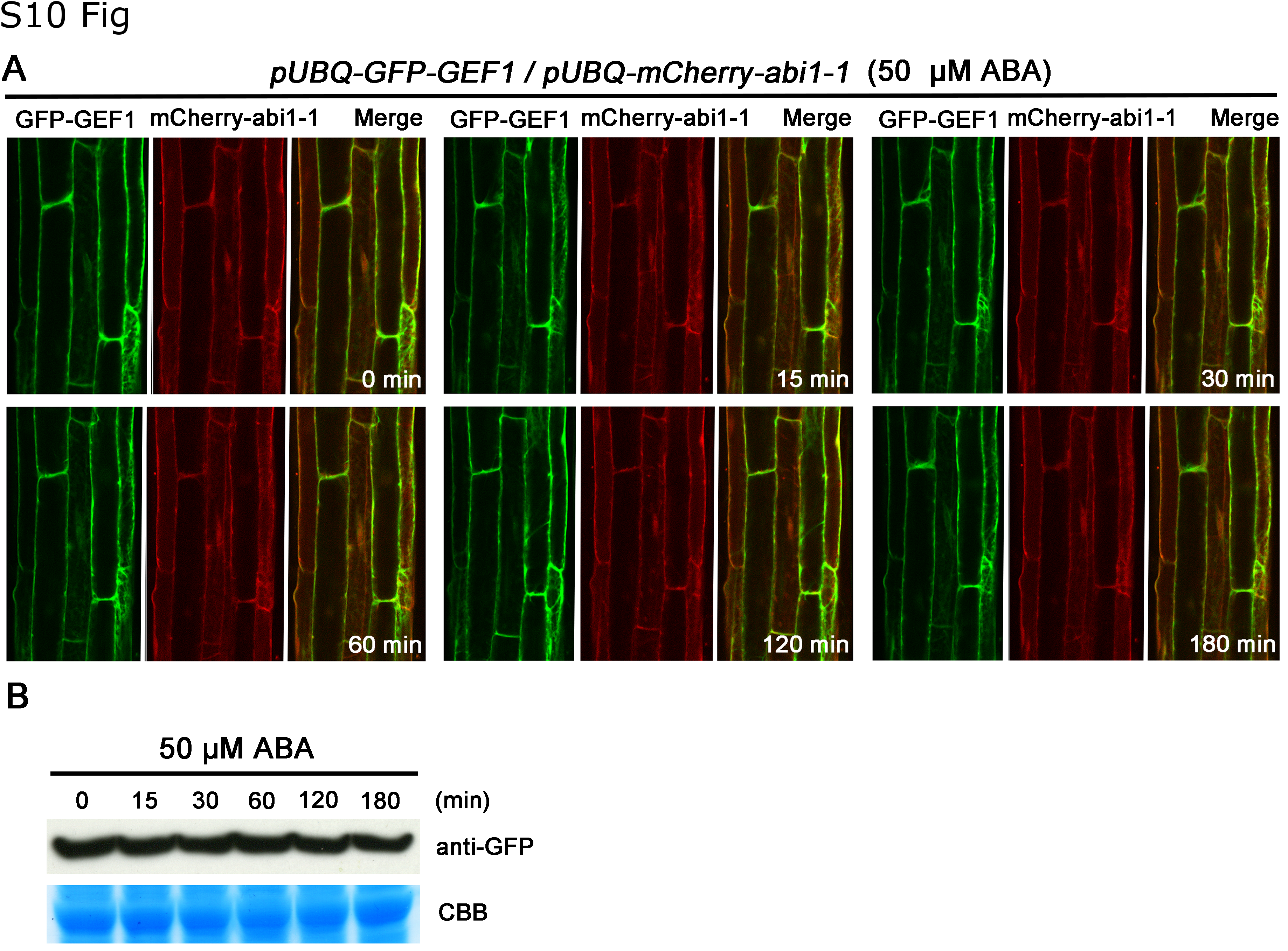

Supplement: S10 Fig — (A) Subcellular localization of GFP-GEF1 in response to ABA in Arabidopsis expressing both GFP-GEF1 and mCherry-abi1-1. Time after ABA treatment is indicated in the Merge images. (B) Immuno-blotting analyses of GFP-GEF1 protein levels in expression lines of both GFP-GEF1 and mCherry-abi1-1. Ten-day-old Arabidopsis seedlings were treated with 50 μM ABA for the indicated times. Total protein was extracted and immune-blotting was carried out with GFP antibody. Coomassie blue staining of SDS-polyacrylamide gel (SDS-PAGE) was used as a loading control. (TIF) [file pbio.1002461.s011.tif]

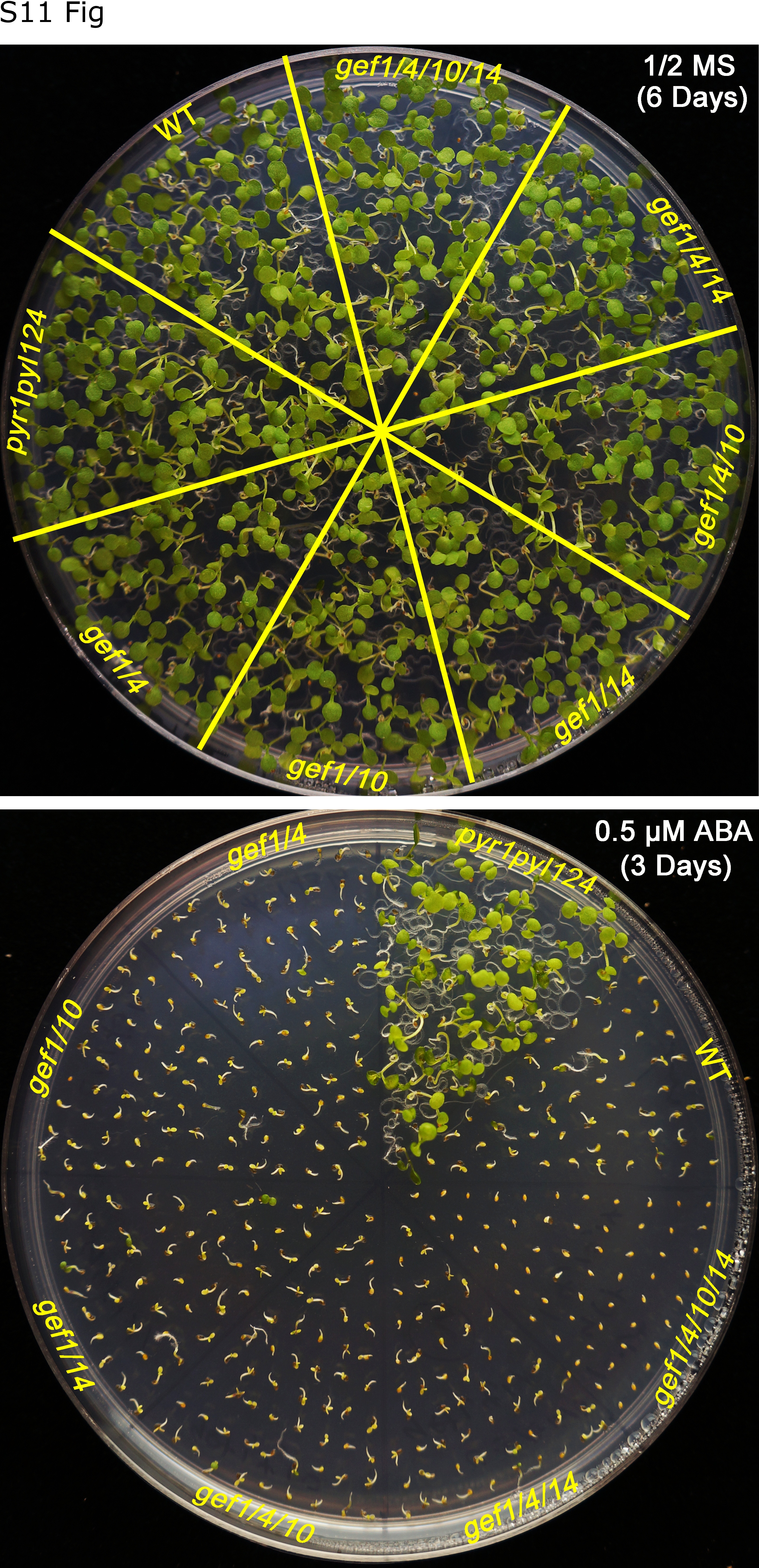

Supplement: S11 Fig — Images of germinating seeds grown on 1/2 MS medium for 6 d (top) or on 1/2 MS media supplemented with 0.5 μM ABA for 3 d (bottom). The depicted plates are the same plate as magnified images shown in Fig 7F. (TIF) [file pbio.1002461.s012.tif]
